# No evidence for modulation of the readiness potential by respiratory phase during natural breathing

**DOI:** 10.1371/journal.pbio.3003982

**Published:** 2026-09-02

**Authors:** Lucas Jeay-Bizot, Raniyah Chishti, Uri Maoz, Aaron Schurger

**Affiliations:** 1 Laboratory for Understanding Consciousness, Intentions, and Decisions in Brains and Machines (LUCID), Chapman University, Orange, California, United States of America; 2 Schmid College of Science and Technology, Chapman University, Orange, California, United States of America; 3 Department of Neurosurgery, Cedars-Sinai Medical Center, Los Angeles, California, United States of America; 4 Fowler School of Engineering, Chapman University, Orange, California, United States of America; 5 Department of Psychology, Chapman University, Orange, California, United States of America; 6 Anderson School of Management, University of California Los Angeles, Los Angeles, California, United States of America; 7 Division of Biology and Bioengineering, California Institute of Technology, Pasadena, California, United States of America; 8 Institut national de la santé et de la recherche médicale (INSERM), Cognitive Neuroimaging Unit, NeuroSpin Center, Gif sur Yvette, France; Aarhus University, DENMARK

## Abstract

Respiratory processes are increasingly implicated in shaping neural activity and behavior. Recent studies have reported a coupling between respiratory phase and the cortical readiness potential (RP), suggesting that breathing may modulate the neural processes preceding voluntary action. Here, using electroencephalography recordings in humans, we re-examine this claim using the original dataset and a new independent dataset, as well as in simulated data with no coupling. We show that the reported association arises from a confound: both RP amplitude and respiratory phase are coupled to movement onset. The original analysis does not control for this dependency, leading to a spurious effect that is also observed in simulated data. When trials are instead grouped by respiratory phase at the time of movement, thereby controlling for this confound, the apparent coupling disappears. Across datasets, Bayesian analyses provide evidence for the absence of an effect under natural breathing conditions. These findings indicate that respiratory phase does not directly modulate RP amplitude during spontaneous behavior. More broadly, they reveal a shortcoming of phase-amplitude coupling analyses applied to epoched data with slowly varying signals, and highlight the importance of controlling for shared dependencies when interpreting physiological–neural relationships.

## Abstract 2

Respiration influences neuronal activity in a number of contexts, and recent work suggests that respiratory phase is coupled to the cortical readiness potential. This study challenges this link and reports that the previously identified association arises from movement onset confounds, highlighting the importance of controlling for shared dependencies when interpreting physiological–neural relationships.

## Introduction

When initiating movements at one’s own pace, unprompted by external cues, the brain’s electrical activity recorded over the scalp reveals a slow ramping activity that begins up to one full second or more in advance of the movement’s onset [1]. Importantly, this ramping begins before one’s own awareness of one’s decision to move, even when deciding spontaneously [2]. The mechanisms giving rise to this signal, the readiness potential (RP), have since been the subject of much scientific debate [3], with more recent interpretations imputing those unconscious processes a facilitatory role rather than a direct causal one [4,5]. In parallel, a growing field of research is reshaping how we understand the influence of gastric, cardiac, respiratory and other physiological processes on brain activity and behavior [6,7].

Recently, these two lines of research converged with reports that both the timing of voluntary self-initiated actions and their reliable neural antecedent, the RP, were coupled to respiratory phase [8,9]. This coupling suggests shared or competing neural processes underlying natural breathing and spontaneous behavior, providing support for the motor competition hypothesis, which posits that respiratory and voluntary motor processes compete for the same resources [8–10], particularly during inhalation [11]. This claim fits broadly within the framework that posits that bodily rhythms constrain cognition [7] and cortical activity [12–14].

The behavioral coupling between natural breathing and self-initiated action was recently reproduced [11], confirming a link between the two processes. However, the nature of this relation remains elusive. A very recent study claims direct evidence for a causal relationship between breathing and self-initiated actions by having human subjects voluntarily modulate their breathing or by initiating their movements during specific phases of breathing [10]. However, respiration can operate both autonomically (“natural breathing”), driven by deep-brain processes in the pre-Bötzinger complex [15], as well as voluntarily through processes in the supplementary motor area (SMA) of the neocortex [16,17], a region widely reported to be involved in self-initiated motor actions [3,18,19]. In particular, invasive recordings in humans have identified different neural structures involved in volitionally-controlled breathing as well as attention to breathing versus natural breathing [20]. It is therefore unsurprising that instructed voluntary breathing might causally modulate self-initiated actions.

Following these recent results of a coupling between cortical activity and natural breathing during self-initiated voluntary action [8,9], the notion of a causal relation between respiration and the RP has been extensively repeated in the literature (see S1 Text) [3,9,13,21–42]. However, to support the claim that respiratory phase has a direct influence on RP amplitude, all reasonable potential confounds must first be ruled out. We show here that a simple confound suffices to explain the observed coupling between respiratory phase and the RP: RP amplitude is also coupled with movement onset, which, as these studies rightly reveal, tends to coincide with breathing phase. A direct relationship between respiration and RP amplitude requires variation in one variable while holding the other constant; the original analysis does not satisfy this condition. Here we show that by grouping RP amplitudes according to respiratory phase (thereby fixing the respiration-behavior coupling), evidence for a direct RP-respiration coupling disappears. We show this to be true in the original dataset [8], in a new independent dataset we collected, and in a simulated dataset expressly engineered to have no direct coupling.

Furthermore, we argue that the recent findings of a causal relation between instructed breathing and voluntary movements [10] do not, on their own, establish a relationship under natural breathing conditions as they fail to address the lack of correlation between natural (uninstructed) respiratory phase and the amplitude of the RP that we demonstrate in this work.

In both original studies [8,9], the RP-respiration coupling was assessed using the modulation index (MI), a tool from the phase-amplitude coupling (PAC) literature [43]. This analysis consisted of grouping EEG amplitudes into bins, within each trial, according to the respiratory phase participants were in within each bin during the 4 s preceding movement initiation. In this analysis, the respiratory phase was divided into six distinct bins (start, middle, and end, for inspiration and expiration). Typically, in any given 4-s-long trial, participants went through all six phases of respiration, yielding six average EEG amplitudes per button press. Critically, most trials ended during expiration (as per the behavioral finding), so the EEG amplitudes for the expiration bins tended to occur just before button press, i.e., when the RP was already known to be maximal – constituting a clear confound for RP-respiration coupling. More broadly, this reveals a general failure mode of PAC analyses applied to epoched data with slowly varying signals [44]. Our findings therefore caution against applying phase-amplitude coupling analyses to slow time-locked signals without appropriate controls, although we have not yet identified any other studies that are affected by this confound.

A more appropriate binning approach and analysis is to group the trials according to the phase of respiration in which the button press occurred and compare the RP amplitudes among these groups. An analysis using this binning approach reveals no effect of respiratory phase on RP amplitude in the original data or in the new data that we collected, even when using the original article’s test statistics [8]. This lack of a significant effect when utilizing the original test statistic was further corroborated by a Bayesian analysis showing moderate evidence for no effect in both datasets. Furthermore, simulated data that we specifically generated to have no RP-respiration coupling demonstrated significant coupling using the original binning analysis method, while correctly revealing no effect with ours.

While the original study did not explicitly claim a direct connection between respiratory phase and RP amplitude, it is implied by the proposed motor competition hypothesis. Furthermore, the field at large has clearly interpreted the results as evidence for a direct connection, if not evidence for a causal relation, rather than an indirect connection via movement onset (see S1 Text). We show that the previously reported RP-respiration coupling is driven by a confounding factor—the tendency to initiate movements during expiration—which accounts for the reported effect [8,9] and challenges the interpretation of involuntary respiration as a causal modulator of the RP. In addition, we address recent work that reports a direct relationship between *voluntary* breathing phase and RP amplitude [10], under the tacit assumption that this relationship also applies to involuntary breathing.

## Results

To test whether respiratory phase modulates the RP independently of respiration-behavior coupling, we recorded EEG and respiratory signals from a new sample of 17 participants completing 100 trials each of the classical Libet paradigm. We further re-analyzed the dataset reported by Park and colleagues (2020; *N* = 52) and also analyzed simulated EEG data containing respiration-behavior coupling in the absence of cortical–respiration coupling.

As in Experiment 2 of Park and colleagues (2020), participants initiated a button press at a time of their own choosing while monitoring a rapidly rotating clock. In our experiment, trials were divided into two blocks of 50 trials each, with block order counterbalanced across participants. In one block, following a random delay (0.5 to 0.8 s uniformly distributed) after movement execution, participants reported the position of the clock hand at the time of their movement (M-time). In the other block, they reported the position of the clock hand when they first became aware of their conscious urge to move (W-time). The waiting time was on average 5.03 ± 1.07 s and the standard deviation 1.49 ± 0.56 s. These behavioral results are consistent with previous studies using the same paradigm [8]. Our data also yielded RP amplitudes, averaged over the last 2 s prior to the button press without a baseline applied, with a mean of −1.78 ± 0.93 µV, similar to −1.40 ± 1.05 µV computed from the original data [8] and also within the range of values reported elsewhere [45].

### Coupling of the timing of self-initiated actions to respiratory phase

**Fig 1:**
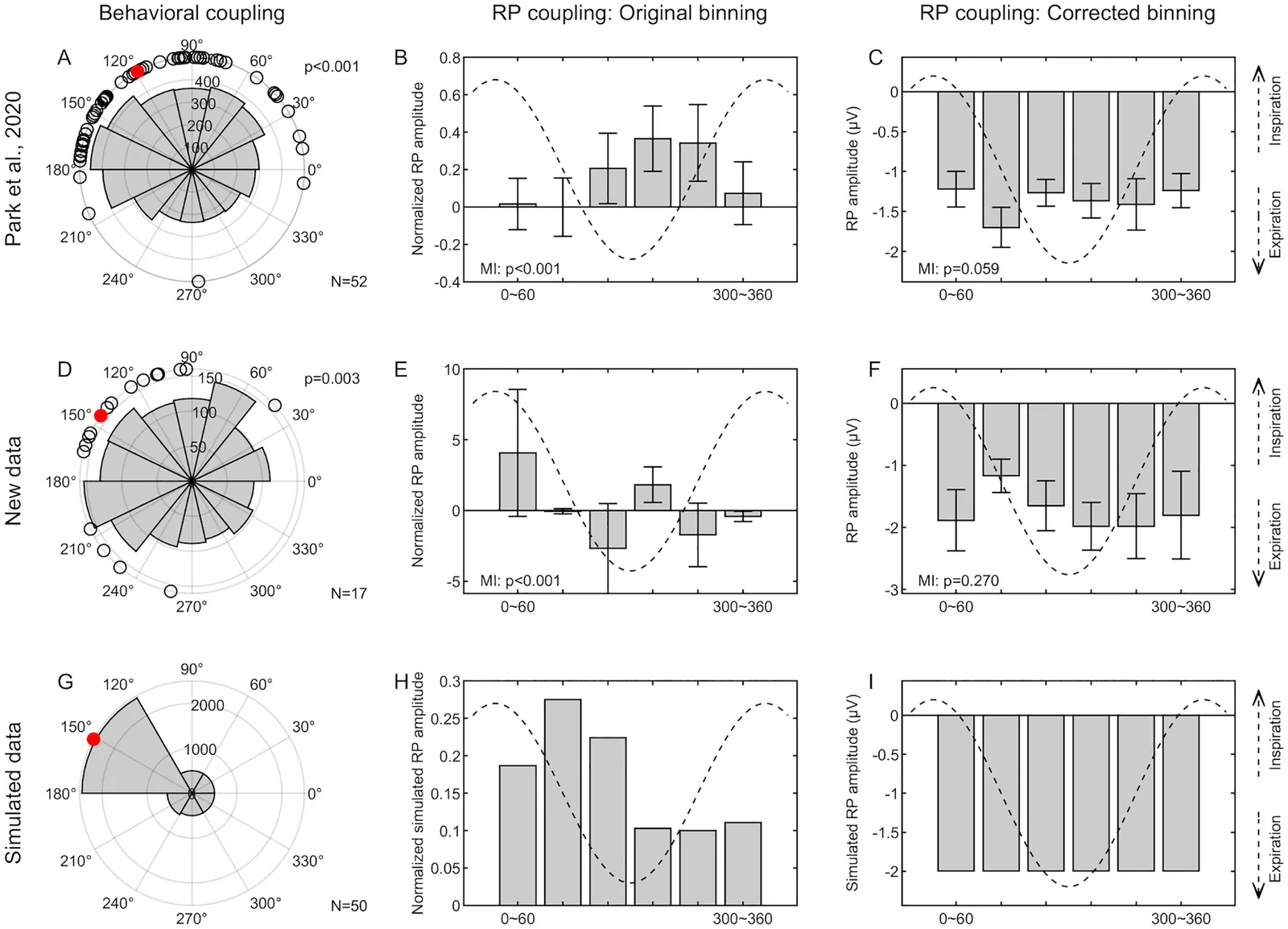
Reproducibility of Park and colleague’s (2020) results across different datasets (pooled data across experiments 1 and 2 from Park and colleagues (2020) in A–C with *n* = 52 participants; our newly collected dataset in D–F with *n* = 17 participants; and simulated data with no RP-respiration coupling in G–I with *n* = 50 pseudo-participants; note that B and E do not display a peak RP at the same location as H, see S3 Text for an explanation) and with different binning methods. The left column (panels A, D, and G) contains radial histograms for all pooled respiratory phases at the time of button press across all participants and trials. The empty circles around the circumference represent the participants’ individual means. The full red circles represent the means across participants’ means. *P*-values for A and D were computed using a one-sided permutation test (Figs 1C, 2C in Park and colleagues 2020). *P*-values for B, C, E and F were computed using a one-sided permutation test identical to Park and colleagues (2020). In particular, the middle column (panels B, E, and H) shows the analysis of Park and colleague’s (2020) data for readiness potential (RP)-respiration coupling (Fig 3B in Park and colleagues (2020). No corrections for multiple comparisons were performed. Individual participants’ values are shown in the supporting information (S5 Fig). No control-group data were collected as the comparison was within participants across phase bins. Normalized RP amplitudes across six equally sized bins of respiratory phase are shown here in panels B, E, and H (± SEM). The right column (panels C, F, and I) displays the RP amplitude as a function of six equally sized bins of respiratory phase using the corrected binning method (±SEM). The data and code used to reproduce this figure are available via OSF and GitHub; some of the original data are available only upon request from the authors of Park and colleagues (2020). See the Data Availability Statement for details.

We first tested the hypothesis that the onset of voluntary movement is associated with natural breathing. We computed the phase of respiration using the instantaneous Hilbert transform [8]. We then used the same permutation-based test as Park and colleagues (2020), to test whether movements occurred uniformly across respiratory phase using the omnibus test for uniformity on circular data [46] and performing random permutations by shifting the respiratory phase. Similarly to Park and colleagues (2020, 2022) and a recent reproduction of this result [11], we found that participants pressed the button more frequently during expiration (Fig 1; permutation test, *p* = 0.003). The mean respiratory phase at the time of the button press occurred during expiration for 13 out of 17 of our participants. However, the coupling occurred slightly later in the breathing cycle in our data (Fig 1D).

We next estimated the effect size of respiration-behavior coupling by measuring the percentage change in the test statistic relative to randomly permuted data (see S2 Text and S1 Fig). This effect was small, amounting to less than 5% in both datasets. The difference in the preferred timing of the coupling was not explained by the inclusion of M-time trials in our data or the difference in number of trials and remained even after aligning to the onset of muscle activity (see S2 Text and S2 Fig). We therefore interpret the small shift in preferred respiratory phase between our dataset and Park and colleagues (2020) as likely reflecting sampling variability or sample-specific characteristics rather than a qualitative difference in respiration–movement coupling. This interpretation is further supported by the small shift observed in Shibata and Ohira (2026), whose reported coupling occurred slightly earlier in the breathing cycle than in Park and colleagues (2020).

### Coupling of RP amplitude to respiratory phase

Next, we tested whether the amplitude of the cortical RP was modulated by respiratory phase. We first used the same MI, test statistic, and binning approach used in previous reports [8,9], which do not account for the coupling between respiration and behavior (original binning). We then used a complementary binning approach based on respiratory phase at the time of movement, thereby equating respiration-behavior coupling across bins (corrected binning).

When EEG data were binned according to respiratory phase within each trial [8,9], we reproduced the previously reported effect in the original dataset (Fig 1B; permutation test, *p* < 0.001) and also observed significant coupling in our new dataset (Fig 1E; permutation test, *p* < 0.001). However, the same effect was also present in simulated data designed to contain respiration-behavior coupling but no respiration–RP coupling (Fig 1H). Moreover, this result was unstable in both the original and new datasets: changing the normalization parameter or applying outlier correction eliminated the significant coupling effect (see S3 Text).

When EEG data were instead binned according to respiratory phase at the time of movement, thereby controlling for behavioral coupling to respiration, we found no significant coupling between RP amplitude and respiratory phase in our new dataset (Fig 1F; permutation test, *p* = 0.270) nor in the original dataset (Fig 1C; permutation test, *p* = 0.059). Simulated data correctly revealed the expected uniform distribution across bins when no coupling was present (Fig 1I). Although the respiration-RP amplitude in the original dataset with our corrected binning approach approached significance, this trend disappeared when outlier rejection was applied (see S3 Text).

### Evidence for no coupling of the amplitude of the RP with natural breathing

The absence of a significant association between respiratory phase and RP amplitude after accounting for the coupling between respiration and behavior does not, by itself, constitute evidence that no coupling exists. We therefore used a Bayesian approach to assess evidence for or against a coupling between respiratory phase and RP amplitude.

Because there is no established Bayesian test for circular data, we instead split the data within each participant into two equal-sized bins and performed a Bayesian paired-samples *t* test. Trials were ranked according to their circular distance from each participant’s median preferred respiratory phase for button presses. The half closest to this phase were grouped as in-phase trials, and the half farthest as out-of-phase trials. If RP amplitude depends on respiratory phase, this balanced split should reflect the expected difference between the two trial types.

**Fig 2:**
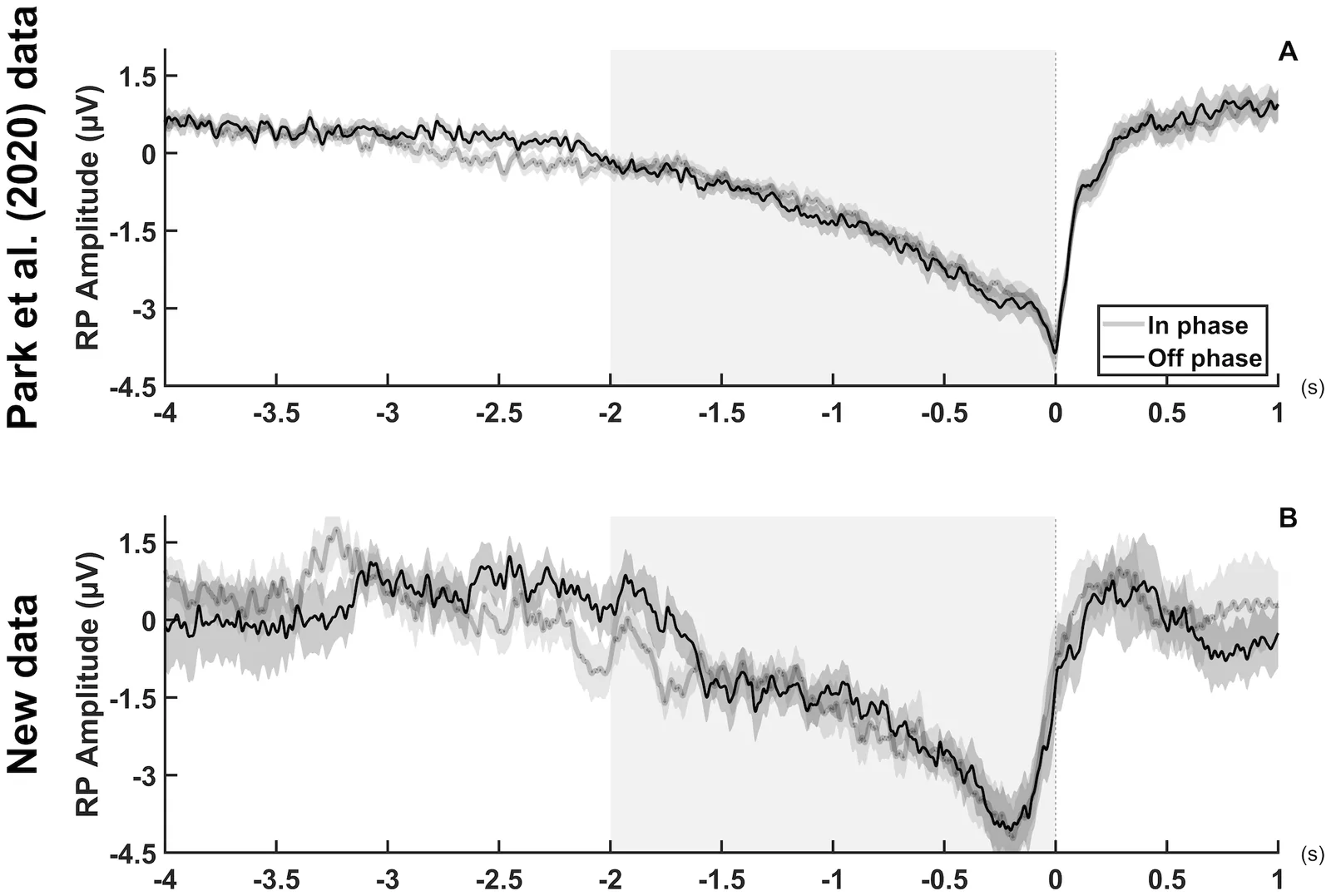
Time course of the readiness potential sorted according to whether t0 (movement onset) occurred during the preferred phase (“in phase”; gray line), or during the least preferred phase of respiration (“off phase”; black line). Shaded regions around signals indicate the SEM. Shaded region from −2 to 0 s indicates the time range used for the Bayesian *t* test. Time 0 is the onset of button press. A Data from Park and colleagues (2020), *n* = 52 participants, BF_01_ = 4.713 (Bayesian paired-samples *t* test). B Data we collected, *n* = 17 participants, BF_01_ = 3.316 (Bayesian paired-samples *t* test). The data and code used to reproduce this figure are available via OSF and GitHub; some of the original data are available only upon request from the authors of Park and colleagues (2020). See the Data Availability Statement for details.

For the original data we found a BF_01_ of 4.713 with a posterior median of 0.110 (95% credible interval [−0.153, 0.375]) which corresponds to moderate evidence for the null, i.e., the two RPs being the same. Similarly, for our data we found a BF_01_ of 3.316 with a posterior median of −0.135 (95% credible interval [−0.584, 0.301]) which also corresponds to moderate evidence for the null. Together, and considering that our split was chosen to target the difference between the two bins in terms of respiration-behavior coupling, this provides moderate evidence that the amplitude of the RP does not depend on the phase of respiration during the button press. Bayesian testing with balanced trials across conditions, thus revealed evidence for no modulation of the RP amplitude by respiratory phase (Fig 2).

As a complementary analysis, to ensure our results did not stem from our binning approach, we also split the trials into unbalanced bins of trials that occurred during expiration versus during inspiration. This split also revealed moderate evidence against a difference. For the original data we found a BF_01_ of 3.965 with a posterior median of −0.136 (95% credible interval [−0.402, 0.128]), which corresponds to evidence for the null. Similarly, for the new data we found a BF_01_ of 3.002 with a posterior median of −0.168 (95% credible interval [−0.620, 0.270]).

## Discussion

Physiological rhythms are increasingly implicated in shaping neural activity and behavior [6,13]. In this context, recent reports of a coupling between respiratory phase and the cortical RP have been interpreted as evidence that breathing modulates the neural processes preceding voluntary action [8,9]. However, establishing such a relationship requires ruling out plausible confounds, particularly the known coupling between respiration and the timing of self-initiated movements. We show in this work that when accounting for such confounds, the data provide evidence against a coupling between respiration and the readiness potential.

Across the original data [8], newly collected data, and simulated data, we show using the original test statistics [8] that the effect disappears once confounds are accounted for (Fig 1) and that there is moderate evidence for no coupling between respiratory phase and the amplitude of the RP using Bayesian statistics (Fig 2).

It is important to note that neither the original study nor our study provides a test for causality. However, we show that the strong coupling between breathing and the RP reported in Park and colleagues (2020) most likely arises from a confound, and that the data lack any direct correlation that would be expected from a causal relation. Only a study using causal tools (e.g., an intervention) can test for causality. Recent work, using instructed (voluntary) breathing, found a relationship between the amplitude of the RP and respiratory phase [10], but such findings do not establish a relationship under natural (involuntary) conditions. Firstly, although correlation does not imply causation, a causal account of modulation by natural breathing would ordinarily be expected to produce some observable covariation between respiratory phase and RP amplitude. Thus, these newer findings raise a key issue: how can a causal relationship be established in the absence of covariation between the putative cause and effect? Secondly, more parsimonious alternative explanations have not been readily ruled out. These include, for example, instructing participants to time actions with specific respiratory phases, which shifts breathing from an involuntary, brainstem-driven process to a voluntary, cortically controlled one [47]. Voluntary breathing is known to recruit prefrontal and supplementary motor networks [20], the very same networks involved in voluntary movement generation [3,48], introducing additional top-down control demands that are themselves capable of modulating preparatory motor activity and RP amplitude. Similarly, attention to breathing without volitional control of breathing also recruits prefrontal and supplementary motor networks [20]. Because the RP is recorded over, and hypothesized to stem from, supplementary motor regions [3], only an intervention that maintains natural breathing can assess causal evidence for a modulation of the RP by natural breathing. Another possibility concerns time pressure. Under natural conditions, expiration lasts substantially longer than inspiration [49,50]. Participants instructed to initiate movements during inspiration may therefore experience increased urgency, as the available time window is shorter and more constrained. Time pressure and urgency are known to influence the amplitude and slope of the RP [5]. This interpretation is further supported by the authors’ own data, which show that “breathe-in” movements are clustered toward the very end of the inspiratory phase, whereas “breathe-out” movements are distributed closer to the middle of expiration [10]. Taken together, these considerations cast substantial doubt on the existence of a direct causal relationship between natural respiratory phase and RP amplitude. At present, the data do not warrant a generalization in the interpretation of the results from instructed to natural breathing. Resolving this issue will require experiments that dissociate breathing from voluntary control and time pressure, as well as analyses that explicitly reconcile causal claims with the absence of correlation under natural breathing conditions.

Furthermore, our findings highlight a serious pitfall in the assumptions of PAC analyses: when used on epoched data in the presence of a slowly varying signal, PAC analyses can yield spurious results. Because the MI is computed over a single epoch of data, the period of the signals being studied needs to be shorter than the length of the epoch in order to ensure all bins are represented. Furthermore, because physiological data are noisy, its period should ideally be much shorter than the duration of the epoch [44]. Here, the original binning approach circumvented this issue by concatenating many trials creating a long epoch of noncontinuous data [8]. However, this introduces a further confound: those epochs were not randomly sampled but conditioned on the occurrence of a movement, which as we showed in this work, can yield spurious findings if the phase of the signal studied is correlated with the conditioned event (here movement onset). Our results call for caution when employing the MI, especially when dealing with slow-varying signals, small epoch length and time-locked data. This observation invites future work to explore other approaches to coupling analyses. This is especially relevant as the field of research into the relationship of bodily rhythms to neural, cognitive, and behavioral processes rapidly expands [6,7,14,26].

Our results raise additional questions regarding the causal role of the RP in self-initiated movements. If the timing of self-initiated movements is influenced by respiratory phase as the behavioral results suggest, and the amplitude of the RP reflects an influence on the timing of the decision, as some models of the RP suggest [4,5], then one ought to find a difference in RP amplitude when grouped by respiratory phase. For instance, the leaky stochastic accumulator model (LSA) proposes that spontaneous self-initiated movements are initiated once a certain threshold of cortical activity has been reached [5]. This process is then reflected in the averaged RP. When the threshold is crossed early, the RP is steeper and vice versa. Another model proposes that the likelihood of a movement being initiated at any given instant depends on the phase of ongoing slow cortical potentials (SCPs) [4]. The greater the ratio of movements occurring during the trough versus the peak of SCPs, the more negative the RP. Interestingly, both models predict that sorting trials according to respiratory phase should yield different RP amplitudes. Because action onset is more likely to occur during expiration, ongoing fluctuations in the EEG should explain behavior slightly less during expiration. When this happens, in some instances, the accumulation process would not have reached a threshold or the coupling of behavior with SCP would be diminished resulting in smaller RPs during expiration.

Both interpretations assume that EEG amplitude and respiration are independent and not both reflections of the same mechanism. This interpretation would have yielded positive results for the resting state analysis in Park and colleagues (2020), so we are setting it aside here, although it is important to note that SCPs have been linked to respiration elsewhere [51]. However, considering the small effect size of the respiration-behavior coupling (see S2 Text), the effect might not be retrievable given the signal-to-noise ratio of physiological data. A future study could investigate this question in more detail with a targeted power analysis.

Another possibility is that respiratory phase does not influence the timing of behavior but simply correlates with it. There could be alternative interpretations here, for instance, when participants are getting ready to press a button their expiration to inspiration (I:E) ratio becomes greater, or maybe their respiratory phase resets at the start of each trial. The authors did account for that possibility by running experiment 3 (see Park and colleagues 2020), however in experiment 3 participants were performing a different type of task (detection and reaction time rather than self-initiated voluntary action) and it is possible that this other type of task does not have the skew in I:E ratio or reset in respiratory phase that a standard self-initiated task might have. We invite future investigations to focus on these possibilities.

It could also be that the RP plays no causal role in behavior. For example, some research has suggested that the RP reflects processes other than movement initiation (e.g., Miller and colleagues 2011 found the clock monitoring was enough for an RP to appear [52]). We invite future research, using interventions, to test the causal role of both natural respiration and the RP on self-initiated action timing, as these findings call into question the causal role of the RP. Causally testing the role of respiration could be done by subliminally entraining respiratory rate, with different I:E ratios or by incentivizing initiating trials only during certain phases of respiration without drawing attention to breathing. Causally testing the role of the cortical sources of the RP on behavior could be done using brain stimulation to modulate their excitability.

While concerns might be raised about our sample size, we had a comparable number of trials per participant as in the original study [8]. Furthermore, our dataset replicates the behavioral effect. Additionally, we had access to both the original dataset and our newly acquired data which both show consistent evidence for the null (Fig 2), indicating that the available data are sufficient to address the question.

Furthermore, the simulated data clearly and sufficiently show that the original analysis erroneously identifies a relationship between RP amplitude and breathing phase (Fig 1H) while the proper binning procedure correctly demonstrates no relationship between respiration and RP amplitude (Fig 1I). Our simulations demonstrate that the apparent effect arises analytically from the binning procedure itself rather than from a true effect or a direct correlation. Bayesian analyses (Fig 2) following a corrected binning approach reveal that there is evidence for no coupling between respiratory phase and the amplitude of the RP in both the full original dataset (*n* = 52 participants) and in a novel independent dataset (*n* = 17 participants).

We additionally explored (S3 and S4 Figs) how normalization and outlier rejection can heavily impact the results. For instance, in Fig 1C the p-value can jump from *p* = 0.059 to *p* = 0.591 after outlier rejection (see S3 Text). This sensitivity points to a broader issue, beyond the scope of this manuscript, regarding researchers’ degrees of freedom over data analysis. The dependence of statistical conclusions on reasonable analytical decisions is a central motivation for open science practices [53]. One solution, recently proposed is multiverse and specification-curve analyses to evaluate the robustness of findings across a range of defensible analytical choices and thereby improve their credibility and replicability [54]. Our findings encourage researchers to adopt open science approaches when assessing the robustness and replicability of their results.

In conclusion, while the data confirm a coupling between respiratory phase and the timing of self-initiated actions (Fig 1A and 1D), contrary to what was previously suggested, there is no evidence that respiratory phase is directly coupled with the RP (Figs 1C, 1F and 2). We show that the purported coupling between the RP and breathing stems from a confound common to both: their respective coupling to movement onset.

## Materials and methods

### Ethics statement

All participants in the present study provided written informed consent. The study was approved by the Chapman University Institutional Review Board (IRB-22-21) and was conducted in accordance with the principles of the Declaration of Helsinki. For the data originally reported by Park and colleagues (2020), ethical approval and written informed consent were obtained as described in that publication.

### Overview of datasets

We analyzed three datasets: (i) the original dataset reported by Park and colleagues (2020; *N* = 52), (ii) a newly collected empirical dataset (*N* = 17), and (iii) a set of simulated data (*N* = 50). All analyses were applied identically across datasets unless otherwise specified. No sex or gender analyses were performed as they did not pertain to the research question. No statistical method was used to determine sample size in advance.

### Original empirical dataset

We received preprocessed data (i) reported by Park and colleagues (2020; *N* = 52) that we re-analyzed. This dataset contains data from two self-initiated button press experiments.

In experiment 1, 20 participants performed a version of Kornhuber and Deecke’s task [1]. They completed three 8-min blocks of self-paced right-index finger button presses roughly every 8–12 s with eyes closed and with white noise as a background auditory input. They were further instructed not to count or press rhythmically.

In experiment 2, 32 participants performed a version of Libet and colleagues’s task [2]. Participants monitored a red dot going around a clock face completing a full revolution every 2.56 s. Participants were instructed to wait one full revolution and then press a button with their right index finger at any time they wanted. Participants were further instructed to avoid pre-planning or pressing at regular intervals. The clock’s hand then disappeared immediately and participants were then asked to report where on the clock the dot was when they first felt the urge to press the button. Successive trials were separated by a random inter-trial interval of 4–8 s. Participants performed 3 blocks of 25 trials for a total of 75 trials each (except for 1 participant who completed 90 trials).

The authors combined those two experiments into one dataset totaling 52 participants. More details are available as reported in Park and colleagues (2020).

### Original data acquisition and preprocessing

In the original study, EEG data were recorded with a 64-channel BioSemi ActiveTwo system at 2,048 Hz and online low-pass filtered at 400 Hz. Respiration was recorded with a Biopac MP36 respiratory belt at 2,000 Hz.

The continuous EEG time series was downsampled to 512 Hz, band-pass filtered between 0.1 and 40 Hz, and re-referenced to the common average. Data were epoched from 4 s before until 1 s after the button press. Trials containing excessive noise, defined in the original analysis as values exceeding three standard deviations, were excluded. No baseline correction was applied.

For each participant, the RP was extracted from the electrode with the largest negative pre-movement RP amplitude, from among electrodes Cz, FCz, Fz, and AFz. Respiratory signals were band-pass filtered between 0.2 and 0.8 Hz, and instantaneous respiratory phase was obtained from the analytic signal using the Hilbert transform. More details are available as reported in Park and colleagues (2020).

### Newly collected empirical dataset

We additionally collected data (ii) using Libet’s (1983) task from 17 participants (3 males, 14 females; age 19–34, mean 23.6, SD 5.0; 2 left-handed, 15 right-handed).

The task was implemented in PsychoPy (v.2020.2.10). Participants monitored a dot (in lieu of the clock hand) completing a full revolution around the clock dial every 2.56 s. Participants were instructed to wait one full revolution and then press a button with their right index finger at any time they wanted. Participants were further instructed to avoid pre-planning or pressing at regular intervals. The dot then disappeared following a 0.5–0.8 s delay (as reported in Libet and colleagues, 1983). After the dot’s disappearance, participants were either asked to report where on the clock the dot was when they first felt the urge to press the button (W-time) or when they physically pressed the button (M-time). The two trial types were administered in blocks of 50 trials, with the order randomized across participants.

Because the main analyses concern the amplitude of the RP and the timing of the button presses relative to respiration, we combined the two trial types in the primary analyses.

### Newly collected data acquisition and preprocessing

EEG data were recorded using an ActiChamp Plus amplifier and a 64-channel EasyCap. Data were sampled at 2,500 Hz and online low-pass filtered at 690 Hz. The online EEG reference was Oz.

Respiration was recorded concurrently using a Brain Products respiratory belt. Surface EMG was recorded from the first dorsal interosseous muscle of the right hand. The EMG ground was placed on the ulnar styloid process, the cathode on the first phalange and the anode on the belly of the first dorsal interosseous muscle.

Preprocessing was performed using the FieldTrip toolbox (v.20240129) for MATLAB (v.2023b). The continuous EEG was downsampled to 512 Hz, band-pass filtered between 0.1 and 40 Hz using FieldTrip’s default filtering parameters, and re-referenced to the common average. Data were epoched from 4 s before until 1 s after the button press. Trials containing excessive noise, defined in the original analysis as values exceeding three standard deviations, were excluded. No baseline correction was applied.

For each participant, the RP was extracted from the electrode with the largest negative pre-movement deflection among Cz, FCz, Fz, and AFz. The electrode with the most negative mean voltage between −2 and 0 s across the retained trials was used for all subsequent RP analyses for that participant.

Respiratory signals were downsampled to 512 Hz and band-pass filtered between 0.2 and 0.8 Hz using FieldTrip’s default third-order Butterworth filter, and instantaneous respiratory phase was obtained from the analytic signal using the Hilbert transform.

EMG signals were band-pass filtered from 20 to 200 Hz using FieldTrip’s default filtering parameters, rectified, and low-pass filtered at 10 Hz again using FieldTrip’s default parameters. The detection threshold for movement onset was set within each trial as the 97.5th percentile during the 2 s preceding the button press. Data were re-aligned to the first crossing within the last 0.5 s before the button press. If the threshold was not crossed during that period, no re-alignment was applied to that trial. This re-alignment was not used in the primary analyses.

### Simulated data

We generated 50 completely identical datasets (devoid of noise), each containing 100 trials with a fixed pseudo-RP consisting of a linear buildup of −4 µV over a 2-s period. The use of 50 datasets was solely to preserve the structure of the analysis pipeline, which operated at the dataset level; the simulations themselves were otherwise identical. The results are, of course, identical regardless of the *N* in the simulated dataset. We only varied the proportion of trials terminating at the end of expiration (50% of trials ended at end of expiration; 10% for each of the other five bins). Respiratory phase was a simple linear signal gradually increasing from −π to π cyclically over a 4 s period. Simulations were designed to contain no coupling between respiratory phase and RP amplitude.

### Respiration-behavior coupling analyses

Following the approach of Park and colleagues (2020), we first tested whether movement onset was uniformly distributed across the respiratory cycle. For each participant, respiratory phase was sampled at each button press and tested for circular uniformity using the Hodges–Ajne omnibus test [46], as implemented in the Circular Statistics Toolbox (v.1.21.0.0). The Hodges–Ajne statistic, M, is the minimum number of observations contained in any semicircle; smaller values indicate a stronger departure from circular uniformity.

To account for differences in the durations of inspiration and expiration and for the temporal autocorrelation of respiration, statistical significance was evaluated using phase-shifted surrogate data. Within each participant, the respiratory phase time series was circularly shifted by a random offset while the movement times remained unchanged. The Hodges–Ajne statistic was recalculated for each surrogate dataset. This process was repeated 1,000 times.

For the group-level test, the participant-level Hodges–Ajne statistics were summed. The observed sum was compared with the distribution of summed surrogate statistics. Because stronger nonuniformity produces smaller values, the permutation *p*-value was calculated as the proportion of surrogate statistics smaller than the observed statistic.

### Respiration/RP-amplitude coupling analyses

#### Original binning.

The original binning approach was identical to that used in Park and colleagues (2020). Within each trial, EEG amplitude and respiratory phase in the 4 s preceding the button press were paired at each time point. The paired EEG and respiratory samples were then concatenated across trials within each participant. The EEG samples were assigned to 1–6 respiratory phase bins according to their concurrent phase (start, middle and end of both inspiration and expiration) before computing the mean EEG amplitude within each bin. Note here that time samples taken from the end of expiration are more likely to be paired with EEG values taken very close to movement onset, when the RP is maximal.

To reproduce the normalized phase-amplitude plot from Park and colleagues (2020), mean EEG amplitude was also computed separately for each of the six phase bins within each trial. Trials that did not contain observations in all six bins were excluded from this visualization. The six values within each retained trial were divided by their sum. Since EEG amplitudes can have both positive and negative values, some values were disproportionately large when the sum was near zero, however this step was retained to identically reproduce the visualizations from Park and colleagues (2020) analysis (see S3 Text for a discussion of the normalization procedure). Normalized values were then averaged across trials and then across participants.

#### Corrected binning.

For the corrected binning, we grouped trials according to the phase of respiration at the time of the button press into the six respective bins described above and averaged the RP amplitudes over the 2 s before button press within each of those bins. This approach isolates the effect of respiratory phase at movement onset, while controlling for the known temporal coupling between RP amplitude and time-to-movement. Each bin equivalently contains EEG data spanning the entire 2-s window preceding movement onset. The 2-s window size was selected to encompass most of the negative slope of the RP in both datasets (see Fig 2). We then averaged across participants. We used the same six bins as in the previous approach for respiration (start, middle and end of both inspiration and expiration). We did not perform any normalization.

### Statistical analyses

#### Modulation index analyses.

For the computation of the MI permutation test statistic (*p*-values displayed in Fig 1) we used the exact same approach as described in Park and colleagues (2020; refer to the original article for a full description). This approach is modeled from the PAC MI [44], which is known not to work well with signals with periods close to the size of the analysis window [44] which is the case for epoched EEG RP data. Furthermore, because this approach is meant to be used on the envelope of the amplitude of a positive-going signal [44], for each participant, a constant of 500 was added to each of the six mean EEG amplitudes, following the original analysis code from Park and colleagues (2020), to ensure that all values were positive before normalizing by dividing each bin value by the sum over the bins (see S3 Text). To compute the MI, first the product between the normalized amplitude values and their natural logarithm is taken, then those products are summed together. The natural logarithm of the number of bins, here six, is then added to this sum. The resulting value is then divided by the natural logarithm of the number of bins. This operation results in the MI value ranging from 0 to 1. A value of 0 means perfect uniform distribution across bins, while a value closer to 1 indicates a complete loading of the distribution onto a single bin.

After computing the MI, we generated 1,000 surrogate datasets by circularly shifting the concatenated respiratory phase data by a random offset, and re-computing a surrogate MI. Participant-level MIs were summed to obtain a group statistic, and the same summation was applied to each permutation. Because a greater MI indicates a greater coupling, we computed the p-value as the proportion of surrogate MIs that were greater than the observed MI.

#### Bayesian tests.

A nonsignificant permutation test does not by itself provide evidence in favor of the absence of respiratory modulation. We therefore performed Bayesian analyses to quantify evidence for or against a difference in RP amplitude between trials occurring near to and far from each participant’s preferred movement-related respiratory phase.

Since there is no Bayesian equivalent for the MI permutation test, and since statistics on circular data can violate assumptions of ANOVAs and other standard tests, for Bayesian analysis we split the data into two equally sized bins within each participant. For each participant individually we first found the circular median of their preferred respiratory phase at time of movement onset and grouped together the RPs of the 50% of trials with movements occurring closest to that median. The remaining 50% (those occurring farthest from that median) were gathered into a separate group. This essentially divided the data into RPs occurring during the preferred phase and RPs occurring during the nonpreferred phase. We used the average amplitude over the 2 s preceding the movement and compared it across both bins using a Bayesian two-tailed paired-samples *t* test with an uninformed prior (Cauchy with scale 0.707) using JASP (version 0.18.3) to evaluate evidence for or against an effect. We report the Bayes factor in favor of the null hypothesis, BF_01_, together with the posterior median standardized effect size and its 95% credible interval. The balanced split was selected to specifically target the separation in respiratory phase between the two conditions while preventing differences in the precision of the RP estimates caused by unequal trial numbers.

As a complementary analysis, trials were divided according to whether movement onset occurred during inspiration or expiration. Unlike the median-based split, this comparison preserved the natural imbalance in trial numbers arising from the tendency for participants to move more frequently during expiration. For each participant, RP amplitude was averaged from −2 to 0 s separately for inspiration and expiration trials. These participant-level values were compared using the same two-sided Bayesian paired-samples *t* test and default Cauchy prior described above.

## Supporting Information

S1 Text Impact on the field. (DOCX)

S1 Table Table of the 31 citations of the 26 papers referring to Park and colleagues (2020) EEG results up to November 2024. (DOCX)

S2 Text Difference in phase of behavioral results. (DOCX)

S1 Fig Circular histograms showing proportions of the pooled distribution of respiratory phase for randomly sampled events (dark gray) and events locked to the button press (light gray) for both Park and colleagues (2020) dataset (A; *n* = 4,539, trials) and our new dataset (B; *n* = 1,587, trials) across all trials. Expiration ranges from 0° to 180° and inspiration from 180° to 360°. The data and code used to reproduce this figure are available via OSF and GitHub; some of the original data are available only upon request from the authors of Park and colleagues (2020). See the Data Availability Statement for details.
(TIFF)

S2 Fig Circular histograms showing distribution of the pooled respiratory phases locked to the button presses. White circles are participants circular means. Red circles are circular means across participants’ circular means. Expiration ranges from 0° to 180° and inspiration from 180° to 360° for *n* = 17 participants. A data for W-time trials only. B data for M-time trials only. C data for the first 75 trials only. D data for all trials realigned to EMG onset. The data and code used to reproduce this figure are available via OSF and GitHub; some of the original data are available only upon request from the authors of Park and colleagues (2020). See the Data Availability Statement for details.
(TIFF)

S3 Text Outliers and normalization. (DOCX)

S3 Fig Impact of outliers on the amplitude of the RP as a function of six equally sized bins of the respiratory phase using the original binning method for *n* = 52 participants. Error bars represent the SEM. A No outliers rejected. B One single most extreme outlier trial rejected. C One single most outlier participant rejected. The data and code used to reproduce this figure are available via OSF and GitHub; some of the original data are available only upon request from the authors of Park and colleagues (2020). See the Data Availability Statement for details.
(TIFF)

S4 Fig Impact of normalization on the amplitude of the RP as a function of six equally sized bins of the respiratory phase using the original binning method (top row) and the corrected binning method (bottom row) for *n* = 52 participants. Error bars represent the SEM. A Sum normalization with original binning, identical to Park and colleagues (2020) Fig 3B. B No normalization with original binning. C Sum normalization with corrected binning. D No normalization with corrected binning. The data and code used to reproduce this figure are available via OSF and GitHub; some of the original data are available only upon request from the authors of Park and colleagues (2020). See the Data Availability Statement for details.
(TIFF)

S5 Fig Reproduction of Fig 1 with individual data points. Reproducibility of Park and colleagues 2020’s results across different datasets (pooled data across experiments 1 and 2 from Park and colleagues (2020) in A–C with *n* = 52 participants, our newly collected dataset in D–F with *n* = 17 participants, and simulated data with no RP-respiration coupling in G-I with *n* = 50 pseudo-participants) and with different binning methods. The first column (panels A, D, and G) contains radial histograms for all pooled respiratory phases at the time of button press across all participants and trials. The empty circles around the circumference represent the participants’ individual means. The full red circles represent the means across participants’ means. *P*-values for A and D were computed using a one-sided permutation test (Figs 1C, 2C in Park and colleagues 2020). Empty circles in the second and third columns represent individual participants’ means. *P*-values for B, C, E, and F were computed using a one-sided permutation test identical to Park and colleagues (2020). No corrections for multiple comparisons were performed. No control-group data were collected as the comparison was within participants across phase bins. Normalized RP amplitude as a function of six equally sized bins of respiratory phase is shown (±SEM). The third column (panels C, F, and I) displays the RP amplitude as a function of six equally sized bins of respiratory phase using the corrected binning method (±SEM). The data and code used to reproduce this figure are available via OSF and GitHub; some of the original data are available only upon request from the authors of Park and colleagues (2020). See the Data Availability Statement for details.
(TIFF)

## Abbreviations

MI: modulation index
PAC: phase-amplitude coupling
RP: readiness potential
SCPs: slow cortical potentials
SMA: supplementary motor area
LSA: leaky stochastic accumulator model.
