# Supplementary material for: No evidence for modulation of the readiness potential by respiratory phase during natural breathing
Source: PLoS Biol. 2026 Sep 2;24(9):e3003982. doi: 10.1371/journal.pbio.3003982 (PMC13561358; doi:10.1371/journal.pbio.3003982)
Supplement: Supplementary file 1 [file pbio.3003982.s012.pdf]

From:
Nithya Venkatesan
Publications Assistant

Journal Requirements:

1) Thank you for stating "All participants provided written informed consent. The study was approved by Chapman University's Institutional Review Board"

Please insert an Ethics Statement at the beginning of your Methods section, under a subheading 'Ethics Statement' and include the details.
NOTE: It must include:

i) The name(s) of the Institutional Review Board(s) or Ethics Committee(s)

The name of the institutional review board, "Chapman University's Institutional review board", is stated.

ii) The approval number(s), or a statement that approval was granted by the named board(s)

We have now added the IRB number.

"The study was approved by Chapman University's Institutional Review Board (IRB-22-21) [...]" under **Materials and Methods, Newly collected empirical dataset** section

iii)A statement that formal consent was obtained (must state whether verbal/written) OR the reason consent was not obtained (e.g. anonymity). NOTE: If child participants, the statement must declare that formal consent was obtained from the parent/guardian.]

We have now added a statement that written informed consent was obtained.

"All participants provided written informed consent. The study was [...] conducted in accordance with the principles laid out in the Declaration of Helsinki" under **Materials and Methods, Newly collected empirical dataset** section

2) We ask that a manuscript source file is provided at Revision. Please upload your manuscript file as a .doc, .docx, .rtf or .tex.

NOTE: If manuscript is a LaTeX submission, Please ensure that you provide a single, cohesive .tex source file for your LaTeX revision. You may upload this file as the item type 'LaTeX Source File.' Please also ensure that you are making any formatting changes to both your .tex file and the PDF of your manuscript. If you have any questions, please contact customercare@plos.org. You can find our LaTeX guidelines here:

https://nam11.safelinks.protection.outlook.com/?url=https%3A%2F%2Fjournals.plos.org%2Fplosbiology%2Fs%2Flatex&data=05%7C02%7Cjeaybizot%40chapman.edu%7C43661fcc51dc4f2bbf5e08dec5149934%7C809929af2d2545bf9837089eb9cfbd01%7C0%7C0%7C639164889434384243%7CUnknown%7CTWFpbGZsb3d8eyJFbXB0eU1hcGkiOnRydWUsIlYiOiIwLjAuMDAwMCIsIlAiOiJXaW4zMiIsIkFOIjoiTWFpbCIsIldUIjoyfQ%3D%3D%7C0%7C%7C%7C&sdata=zCxiQO6CV6B2cKn1Co3L8FCB0OhRrc%2Fx5EOzyqDsKR8%3D&reserved=0

Files are provided as .docx

3) Please provide separate figure files in .tif or .eps format. For more information about figure files please see our guidelines:

https://nam11.safelinks.protection.outlook.com/?url=https%3A%2F%2Fjournals.plos.org%2Fplosbiology%2Fs%2Ffigures&data=05%7C02%7Cjeaybizot%40chapman.edu%7C43661fcc51dc4f2bbf5e08dec5149934%7C809929af2d2545bf9837089eb9cfbd01%7C0%7C0%7C639164889434416271%7CUnknown%7CTWFpbGZsb3d8eyJFbXB0eU1hcGkiOnRydWUsIlYiOiIwLjAuMDAwMCIsIlAiOiJXaW4zMiIsIkFOIjoiTWFpbCIsIldUIjoyfQ%3D%3D%7C0%7C%7C%7C&sdata=Wa0OurMfuM7WvN%2FiSDH6eM8BJtU7zZJbznscTXD7UJU%3D&reserved=0
https://nam11.safelinks.protection.outlook.com/?url=https%3A%2F%2Fjournals.plos.org%2Fplosbiology%2Fs%2Ffigures%23loc-file-requirements&data=05%7C02%7Cjeaybizot%40chapman.edu%7C43661fcc51dc4f2bbf5e08dec5149934%7C809929af2d2545bf9837089eb9cfbd01%7C0%7C0%7C639164889434436764%7CUnknown%7CTWFpbGZsb3d8eyJFbXB0eU1hcGkiOnRydWUsIlYiOiIwLjAuMDAwMCIsIlAiOiJXaW4zMiIsIkFOIjoiTWFpbCIsIldUIjoyfQ%3D%3D%7C0%7C%7C%7C&sdata=mbMQ8%2FXj3ivlanD4w8ak0%2F7Ni7KlTeV2YfNj9dCm8Fk%3D&reserved=0

Main figures are provided as .tif

4) Please upload a copy of Figure [1,2] which you refer to in your text on the manuscript file. Or, if the figure is no longer to be included as part of the submission please remove all reference to it within the text.

Copy provided (Fig1.tif; Fig2.tif)

5) We notice that your [supplementary materials] are included in the manuscript file. Please remove them and upload them with the file type 'Supporting Information'. Please ensure that each Supporting Information file has a legend listed in the manuscript after the references list.

SI has been added. A legend has also been added to the main after the references.

6) Thank you for stating "The original data is available by contacting the authors of the original study Park et al. (2020)directly."

In your Data Availability Statement, please ensure you have provided all necessary access information in complete sentences. Access information should include the name of any data repositories used as well as digital object identifiers (DOIs), accession numbers or codes, or other persistent identifiers for all relevant data.

We have now reworded our data accessibility statement to the following:

"The raw data generated in this study are publicly available in the Open Science Framework (OSF) repository at https://doi.org/10.17605/OSF.IO/3CVYK. The processed data and simulated datasets are publicly available through the Zenodo archive of the associated GitHub repository at https://doi.org/10.5281/zenodo.17715626.
The original dataset reported by Park et al. (2020) is not hosted by the authors of the present study. Access to these data may be requested directly from the authors of Park et al. (2020). All code used to process and analyze the data and generate the figures is publicly available through the Zenodo archive of the associated GitHub repository at https://doi.org/10.5281/zenodo.17715626." under the **Data Availability** section

7) Please refer to the following article for PLOS Biology's best-practice recommendations for data presentation:
https://nam11.safelinks.protection.outlook.com/?url=https%3A%2F%2Fjournals.plos.org%2Fplosbiology%2Farticle%3Fid%3D10.1371%2Fjournal.pbio.1002128&data=05%7C02%7Cjeaybizot%40chapman.edu%7C43661fcc51dc4f2bbf5e08dec5149934%7C809929af2d2545bf9837089eb9cfbd01%7C0%7C0%7C639164889434455933%7CUnknown%7CTWFpbGZsb3d8eyJFbXB0eU1hcGkiOnRydWUsIlYiOiIwLjAuMDAwMCIsIlAiOiJXaW4zMiIsIkFOIjoiTWFpbCIsIldUIjoyfQ%3D%3D%7C0%7C%7C%7C&sdata=oymt%2F8qoXoJf3TC5IjLhcKUzjAAiNHae5NLwChc67wQ%3D&reserved=0.
Guidelines for statistical reporting, if relevant, may be found here:
https://nam11.safelinks.protection.outlook.com/?url=https%3A%2F%2Fjournals.plos.org%2Fplosbiology%2Fs%2Fbest-practices-in-research-reporting%23loc-statistical-reporting&data=05%7C02%7Cjeaybizot%40chapman.edu%7C43661fcc51dc4f2bbf5e08dec5149934%7C809929af2d2545bf9837089eb9cfbd01%7C0%7C0%7C639164889434473928%7CUnknown%7CTWFpbGZsb3d8eyJFbXB0eU1hcGkiOnRydWUsIlYiOiIwLjAuMDAwMCIsIlAiOiJXaW4zMiIsIkFOIjoiTWFpbCIsIldUIjoyfQ%3D%3D%7C0%7C%7C%7C&sdata=SdqWwzhEzYU2eA2L7nRCSTUpjgUZ99jQEdHnQmBBKaE%3D&reserved=0.

Because our main figures directly address Park et al. (2020) we preferred to maintain the same data presentation format to facilitate comparison of the results and visualization. We had however added an additional figure with individual data points (Fig. S5).

8) Some material included in your submission may be copyrighted. According to PLOS's copyright policy, authors who use figures or other material (e.g., graphics, clipart, maps) from another author or copyright holder must demonstrate or obtain permission to publish this material under the Creative Commons Attribution 4.0 International (CC BY 4.0) License used by

PLOS journals. Please closely review the details of PLOS's copyright requirements here: PLOS Licenses and Copyright. If you need to request permissions from a copyright holder, you may use PLOS's Copyright Content Permission form.

Please respond directly to this email and provide any known details concerning your material's license terms and permissions required for reuse, even if you have not yet obtained copyright permissions or are unsure of your material's copyright compatibility. Once you have responded and addressed all other outstanding technical requirements, you may resubmit your manuscript within Editorial Manager.

Potential Copyright Issues:

[Figure 1, Figure S5]: Thank you for stating "Please clarify whether these figures were reproduced from a previously published article"

NOTE: If reproduced from a previously published article, please provide written permission from the copyright holder to publish this under our CC BY 4.0 license, or remove the figure / replace the image. Please note we do not recommend using standard request forms available on Publishers' websites, as they grant single use rather than republication under an open access license.

Figures 1 and S5 were not reproduced from a previously published article by copying or reusing figure panels or image files from Park et al. (2020). Instead, we generated these figures ourselves from the same underlying data using our own analysis code. Therefore, all figures in this manuscript were produced by us.

Because the purpose of these figures was to allow direct comparison with the original findings, and because the underlying data are the same, the resulting figures are intentionally very similar to the corresponding figures in Park et al. (2020). We also used a similar presentation style, for ease of comparison.

The underlying data were shared with us directly by the authors following our request. Park et al. (2020) was published in Nature Communications, whose data-sharing policy requires original research articles to include a data availability statement and expects the corresponding or otherwise designated authors to be responsible for materials availability unless otherwise stated. Consistent with that policy, the data availability statement in Park et al. (2020) indicated that the relevant data were available from the authors upon reasonable request. The authors provided the data to us in response to such a request. However, they did not provide a separate written license or explicit publication permission for the dataset beyond sharing the data with us. We have cited Park et al. (2020) appropriately in the manuscript and figure legends.

From:
Lucas Smith
Senior Editor

Your revisions should address the specific points made by each reviewer. Please submit the following files along with your revised manuscript:

1. A 'Response to Reviewers' file - this should detail your responses to the editorial requests, present a point-by-point response to all of the reviewers' comments, and indicate the changes made to the manuscript.
*NOTE: In your point-by-point response to the reviewers, please provide the full context of each review. Do not selectively quote paragraphs or sentences to reply to. The entire set of reviewer comments should be present in full and each specific point should be responded to individually. You should also cite any additional relevant literature that has been published since the original submission and mention any additional citations in your response.

2. In addition to a clean copy of the manuscript, please also upload a 'track-changes' version of your manuscript that specifies the edits made. This should be uploaded as a "Revised Article with Changes Highlighted " file type.

Resubmission Checklist
When you are ready to resubmit your revised manuscript, please refer to this resubmission checklist:
https://nam11.safelinks.protection.outlook.com/?url=https%3A%2F%2Fplos.io%2FBiology_Checklist&data=05%7C02%7Cjeaybizot%40chapman.edu%7C36975427883444814e2508dec349f42e%7C809929af2d2545bf9837089eb9cfbd01%7C0%7C0%7C639162919580728758%7CUnknown%7CTWFpbGZsb3d8eyJFbXB0eU1hcGkiOnRydWUsIlYiOiIwLjAuMDAwMCIsIlAiOiJXaW4zMiIsIkFOIjoiTWFpbCIsIldUIjoyfQ%3D%3D%7C0%7C%7C%7C&sdata=HGYPBP%2F0ok6gQ0DaA5yvfaHSAR5x8c93b6cnKR6Zb60%3D&reserved=0

To submit a revised version of your manuscript, please go to https://nam11.safelinks.protection.outlook.com/?url=https%3A%2F%2Fwww.editorialmanager.com%2Fpbiology%2F&data=05%7C02%7Cjeaybizot%40chapman.edu%7C36975427883444814e2508dec349f42e%7C809929af2d2545bf9837089eb9cfbd01%7C0%7C0%7C639162919580753110%7CUnknown%7CTWFpbGZsb3d8eyJFbXB0eU1hcGkiOnRydWUsIlYiOiIwLjAuMDAwMCIsIlAiOiJXaW4zMiIsIkFOIjoiTWFpbCIsIldUIjoyfQ%3D%3D%7C0%7C%7C%7C&sdata=2%2FnNLFaJmfM%2FJ44r1D2zZlck1mgkQWn8ZIm3Lbb99E8%3D&reserved=0 and log in as an Author. Click the link labelled 'Submissions Needing Revision' where you will find your submission record.

Please make sure to read the following important policies and guidelines while preparing your revision:

Published Peer Review

Please note while forming your response, if your article is accepted, you may have the opportunity to make the peer review history publicly available. The record will include editor decision letters (with reviews) and your responses to reviewer comments. If eligible, we will contact you to opt in or out. Please see here for more details: https://nam11.safelinks.protection.outlook.com/?url=https%3A%2F%2Fblogs.plos.org%2Fplos%2F2019%2F05%2Fplos-journals-now-open-for-published-peer-review%2F&data=05%7C02%7Cjeaybizot%40chapman.edu%7C36975427883444814e2508dec349f42e%7C809929af2d2545bf9837089eb9cfbd01%7C0%7C0%7C639162919580770979%7CUnknown%7CTWFpbGZsb3d8eyJFbXB0eU1hcGkiOnRydWUsIlYiOiIwLjAuMDAwMCIsIlAiOiJXaW4zMiIsIkFOIjoiTWFpbCIsIldUIjoyfQ%3D%3D%7C0%7C%7C%7C&sdata=u%2B8whqwkdZekJOM2zgeXcKnF7onTOxeAAZYkXIvNMh0%3D&reserved=0

PLOS Data Policy
Please note that as a condition of publication PLOS' data policy (https://nam11.safelinks.protection.outlook.com/?url=http%3A%2F%2Fjournals.plos.org%2Fplosbiology%2Fs%2Fdata-availability&data=05%7C02%7Cjeaybizot%40chapman.edu%7C36975427883444814e2508dec349f42e%7C809929af2d2545bf9837089eb9cfbd01%7C0%7C0%7C639162919580791786%7CUnknown%7CTWFpbGZsb3d8eyJFbXB0eU1hcGkiOnRydWUsIlYiOiIwLjAuMDAwMCIsIlAiOiJXaW4zMiIsIkFOIjoiTWFpbCIsIldUIjoyfQ%3D%3D%7C0%7C%7C%7C&sdata=p%2FeXKo0tpGQI3%2Beh5lKwKuiQjqnQxKe9Uox3cFhakdM%3D&reserved=0) requires that you make available all data used to draw the conclusions arrived at in your manuscript. If you have not already done so, you must include any data used in your manuscript either in appropriate repositories, within the body of the manuscript, or as supporting information (N.B. this includes any numerical values that were used to generate graphs, histograms etc.). For an example see here: https://nam11.safelinks.protection.outlook.com/?url=http%3A%2F%2Fwww.plosbiology.org%2Farticle%2Finfo%253Adoi%252F10.1371%252Fjournal.pbio.1001908%23s5&data=05%7C02%7Cjeaybizot%40chapman.edu%7C36975427883444814e2508dec349f42e%7C809929af2d2545bf9837089eb9cfbd01%7C0%7C0%7C639162919580809905%7CUnknown%7CTWFpbGZsb3d8eyJFbXB0eU1hcGkiOnRydWUsIlYiOiIwLjAuMDAwMCIsIlAiOiJXaW4zMiIsIkFOIjoiTWFpbCIsIldUIjoyfQ%3D%3D%7C0%7C%7C%7C&sdata=244UooGOgdefj3ljxecoFu1PzZogD6QTz3qtcdlpbdc%3D&reserved=0

Blot and Gel Data Policy
We require the original, uncropped and minimally adjusted images supporting all blot and gel results reported in an article's figures or Supporting Information files. We will require these files before a manuscript can be accepted so please prepare them now, if you have not already uploaded them. Please carefully read our guidelines for how to prepare and upload this data: https://nam11.safelinks.protection.outlook.com/?url=https%3A%2F%2Fjournals.plos.org%2Fplosbiology%2Fs%2Ffigures%23loc-blot-and-gel-reporting-requirements&data=05%7C02%7Cjeaybizot%40chapman.edu%7C36975427883444814e2508dec349f42e%7C809929af2d2545bf9837089eb9cfbd01%7C0%7C0%7C639162919580827877%7CUnknown%7CTWFpbGZsb3d8eyJFbXB0eU1hcGkiOnRydWUsIlYiOiIwLjAuMDAwMCIsIlAiOiJXaW4zMiIsIkFOIjoiTWFpbCIsIldUIjoyf

Q%3D%3D%7C0%7C%7C%7C&sdata=DLBqbw8FnsnN53D9yKXNUstugRCk4Kdy64zwupqVxDo%3D&reserved=0

Protocols deposition
To enhance the reproducibility of your results, we recommend that if applicable you deposit your laboratory protocols in protocols.io, where a protocol can be assigned its own identifier (DOI) such that it can be cited independently in the future. Additionally, PLOS ONE offers an option for publishing peer-reviewed Lab Protocol articles, which describe protocols hosted on protocols.io. Read more information on sharing protocols at https://nam11.safelinks.protection.outlook.com/?url=https%3A%2F%2Fplos.org%2Fprotocols%3Futm_medium%3Deditorial-email%26utm_source%3Dauthorletters%26utm_campaign%3Dprotocols&data=05%7C02%7Cjeaybizot%40chapman.edu%7C36975427883444814e2508dec349f42e%7C809929af2d2545bf9837089eb9cfbd01%7C0%7C0%7C639162919580845660%7CUnknown%7CTWFpbGZsb3d8eyJFbXB0eU1hcGkiOnRydWUsIlYiOiIwLjAuMDAwMCIsIlAiOiJXaW4zMiIsIkFOIjoiTWFpbCIsIldUIjoyfQ%3D%3D%7C0%7C%7C%7C&sdata=GGRpUBGj3fJlfsw7nGaSowgwlhwyzlk7x6gfhKlkPpc%3D&reserved=0

EDITORIAL REQUESTS:

1) FORMAT: We understand that your original submission needed to be very concise, and that you have kept it that way while submitting to PLOS Biology, given that the previous reviewers were largely supportive of the previous version. While we appreciate your instinct, we do think that the paper should be fleshed out, as this will help the non-familiar reader and will broaden the appeal of your study. Below are a few specific suggestions:

a. Currently the framing of the study and findings are quite deeply embedded in the context of the two papers which your results challenge. However, I think it would be helpful to adjust the introduction and discussion a bit, to provide more of the broader context and background details (for example, what is readiness potential? why is it important? in what other contexts has breathing been linked to neuronal activity, behavior etc? do your findings prompt a re-evaluation of that work too? etc).

We agree with the editor and are thankful for this opportunity to ground our work in the literature. We have now made substantial changes to the introduction in order to introduce the context of the study and background details. We added the following section to introduce the readiness potential:

“When initiating movements at one’s own pace, unprompted by external cues, the brain’s electrical activity recorded over the scalp reveals a slow ramping activity that begins up to one full second or more in advance of the movement’s onset (1). Importantly, this ramping begins before one’s own awareness of one’s decision to move, even when deciding spontaneously (2). The mechanisms giving rise to this signal, the readiness potential (RP), have since been the subject of much scientific debate (3), with more recent interpretations imputing those

unconscious processes a facilitatory role rather than a direct causal one (4, 5). " under the **Introduction** section

We also added the following sections to introduce the broader context of the rapidly expanding field of respiratory to neural coupling in the biological sciences:

"In parallel, a growing field of research is reshaping how we understand the influence of gastric, cardiac, respiratory and other physiological processes on brain activity and behavior (6, 7).

Recently these two lines of research converged with reports that both the timing of voluntary self-initiated actions and their reliable neural antecedent, the RP, were coupled to respiratory phase (8, 9). This coupling suggests shared or competing neural processes underlying natural breathing and spontaneous behavior, providing support for the motor competition hypothesis, which posits that respiratory and voluntary motor processes compete for the same resources (8-10) , particularly during inhalation (11). This claim fits broadly within the framework that posits that bodily rhythms constrain cognition (7) and cortical activity (12-14).

The behavioral coupling between natural breathing and self-initiated action was recently reproduced (11) confirming a link between the two processes. However, the nature of this relation remains elusive. A very recent study claims direct evidence for a causal relationship between breathing and self-initiated actions by having human subjects voluntarily modulate their breathing or by initiating their movements during specific phases of breathing (10). However, respiration can operate both autonomically ("natural breathing"), driven by deep-brain processes in the pre-Bötzinger complex (15), as well as voluntarily through processes in the supplementary motor area (SMA) of the neocortex (16, 17), a region widely reported to be involved in self-initiated motor actions (3, 18, 19). In particular, invasive recordings in humans have identified different neural structures involved in volitionally-controlled breathing as well as attention to breathing versus natural breathing (20). It is therefore unsurprising, that instructed voluntary breathing might causally modulate self-initiated actions." under the **Introduction** section

b. Along these lines, I also found your results and methods sections to be a bit thin on details. As just one example, your results start discussing how 'we reproduced...' - without describing what analysis you did, or in what dataset. As an example from the methods, you discuss how you 'analyzed three datasets' - what types of datasets were these? what type of data? etc... In general, I think both of these sections could be fleshed out to help bring the reader along, and to ensure that people understand what was done and why.

We again thank the editor for this opportunity to better characterize our research. We have now fully fleshed out our **Results** and **Materials and Methods** sections. Please refer to the track changes version as substantial changes have been made to those sections. Figures and results however remain unchanged, we have simply added context and descriptions.

c. I did notice that your SI includes a lot of additional details and discussion, and I wonder if one approach could be to integrate many of those details into the main paper. I think many of the

details that you include in the SI would be reasonable to put in the methods section or in the results and discussion. Please also note that any citations that are included in the SI must also be included in the main manuscript (and reference list), as otherwise these will not be indexed.

We elected to retain discussions of normalization procedures and outlier rejections to the SI in order to maintain a focus on the confound of the behavior-respiration coupling in the main manuscript. Further, for the same reason, we elected to retain the literature search in the SI and we additionally removed the last paragraph from the introduction to that effect as well.

We did however bring the discussion of the causal relationships between respiration, RP and behavior into the Discussion section of the main manuscript.

The following text was moved from SI to the discussion section:

"Our results raise additional questions regarding the causal role of the RP in self-initiated movements. If the timing of self-initiated movements is influenced by respiratory phase as the behavioral results suggest, and the amplitude of the RP reflects an influence on the timing of the decision, as some models of the RP suggest (4, 5), then one ought to find a difference in RP amplitude when grouped by respiratory phase. For instance, the leaky stochastic accumulator model (LSA), proposes that spontaneous self-initiated movements are initiated once a certain threshold of cortical activity has been reached (5). This process is then reflected in the averaged RP. When the threshold is crossed early, the RP is steeper and vice versa. Another model proposes that the likelihood of a movement being initiated at any given instant depends on the phase of ongoing slow cortical potentials (SCPs) (4). The greater the ratio of movements occurring during the trough versus the peak of SCPs, the more negative the RP. Interestingly, both models predict that sorting trials according to respiratory phase should yield different RP amplitudes. Because action onset is more likely to occur during expiration, then ongoing fluctuations in the EEG should explain behavior slightly less during expiration. When this happens, in some instances, the accumulation process would not have reached a threshold or the coupling of behavior with SCP would be diminished resulting in smaller RPs during expiration.

Both interpretations assume that EEG amplitude and respiration are independent and not both reflections of the same mechanism. This interpretation would have yielded positive results for the resting state analysis in Park et al. 2020, so we are setting it aside here, although it is important to note that SCPs have been linked to respiration elsewhere (28). However, considering the small effect size of the respiration-behavior coupling (see S2 Text), the effect might not be retrievable given the signal-to-noise ratio of physiological data. A future study could investigate this question in more detail with a targeted power analysis.

Another possibility is that respiratory phase does not influence the timing of behavior but simply correlates with it. There could be alternative interpretations here, for instance, when participants are getting ready to press a button their expiration to inspiration (I:E) ratio becomes greater, or maybe their respiratory phase resets at the start of each trial. The authors did account for that

possibility by running experiment 3 (see Park et al. 2020), however in experiment 3 participants were performing a different type of task (detection and reaction time rather than self-initiated voluntary action) and it is possible that this other type of task does not have the skew in I:E ratio or reset in respiratory phase that a standard self-initiated task might have. We invite future investigations to focus on these possibilities.

It could also be that the RP plays no causal role in behavior. For example, some research has suggested that the RP reflects processes other than movement initiation (e.g. Miller et al. 2011 found the clock monitoring was enough for an RP to appear (29)). We invite future research, using interventions, to test the causal role of both natural respiration and the RP on self-initiated action timing, as these findings call into question the causal role of the RP. Causally testing the role of respiration could be done by subliminally entraining respiratory rate, with different I:E ratios or by incentivizing initiating trials only during certain phases of respiration without drawing attention to breathing. Causally testing the role of the cortical sources of the RP on behavior could be done using brain stimulation to modulate their excitability."

d. As a last point about fleshing the study out - we understand that since your paper was under consideration at a different journal that several relevant papers have come out on this topic. While we understand that your manuscript already mentions these, we think the discussion of those papers could be expanded a bit, to make sure your findings are presented in the most relevant, and up-to date context.

We added context (see response to 1a) and we additionally expanded on the discussion of Sandoval et al., (2026) in our Introduction section by adding the following:

"The behavioral coupling between natural breathing and self-initiated action was recently reproduced (11) confirming a link between the two processes. However, the nature of this relation remains elusive. A very recent study claims direct evidence for a causal relationship between breathing and self-initiated actions by having human subjects voluntarily modulate their breathing or by initiating their movements during specific phases of breathing (10). However, respiration can operate both autonomically ("natural breathing"), driven by deep-brain processes in the pre-Bötzinger complex (15), as well as voluntarily through processes in the supplementary motor area (SMA) of the neocortex (16, 17), a region widely reported to be involved in self-initiated motor actions (3, 18, 19). In particular, invasive recordings in humans have identified different neural structures involved in volitionally-controlled breathing as well as attention to breathing versus natural breathing (20). It is therefore unsurprising, that instructed voluntary breathing might causally modulate self-initiated actions. "

2) TITLE: After discussing your paper with my colleagues, we did wonder if it would be a good idea to tweak the title slightly, in light of recent literature. If you agree, we would suggest you update the title to:

'No evidence for modulation of the readiness potential by respiratory phase during natural breathing'

^we are happy to discuss further.

We agree that this title change better characterizes the results of our study and have made the change accordingly.

3) ABSTRACT: Please note that it is PLOS Biology policy to clearly state in the abstract what model organisms was examined in the study. Please update the abstract accordingly.

We have added a mention that data was collected from humans in the abstract:

"Here, using electroencephalography recordings in humans, we re-examine this claim using the original dataset and a new independent dataset, as well as in simulated data with no coupling."

4) ETHICS STATEMENT: Please update your ethics statement to include the approval number for your protocol approved by Chapman University's IRB. Please also indicate whether your study was conducted in accordance with the principles laid out in the Declaration of Helsinki.

We have modified the ethics statement to the following:

"The study was approved by Chapman University's Institutional Review Board (IRB-22-21)"
and
"All participants provided written informed consent. The study was [...] conducted in accordance with the principles laid out in the Declaration of Helsinki" under **Materials and Methods, Newly collected empirical dataset** section

5) DATA and CODE: Thank you for providing the raw data on OSF, and the process and simulated data on GitHub (including with a zenodo DOI). These largely meet our requirements, but:

a. please add a sentence to every relevant figure legend, pointing readers to the underlying data.

The following sentence has been added to both main figures captions:

"*The data and code used to reproduce this figure are available via OSF and GitHub; some of the original data are available only upon request from the authors of Park et al. (2020). See the Data Availability Statement for details.*"

b. please update your data availability statement in our editorial manager system, to indicate that the code can also be found on your GitHub deposition.

We have updated the data availability statement:

"*The raw data generated in this study are publicly available in the Open Science Framework (OSF) repository at https://doi.org/10.17605/OSF.IO/3CVYK. The processed data and simulated datasets are publicly available through the Zenodo archive of the associated GitHub repository at https://doi.org/10.5281/zenodo.17715626.*

The original dataset reported by Park et al. (2020) is not hosted by the authors of the present study. Access to these data may be requested directly from the authors of Park et al. (2020).

All code used to process and analyze the data and generate the figures is publicly available through the Zenodo archive of the associated GitHub repository at https://doi.org/10.5281/zenodo.17715626."
