# Supplementary material for: No evidence for modulation of the readiness potential by respiratory phase during natural breathing
Source: PLoS Biol. 2026 Sep 2;24(9):e3003982. doi: 10.1371/journal.pbio.3003982 (PMC13561358; doi:10.1371/journal.pbio.3003982)
Supplement: Supplementary file 2 [file pbio.3003982.s001.docx]

**S1 Text: Impact on the field**

In order to assess the impact this result had on the field, a search was performed using the Google Scholar search engine for articles that cited Park et al. 2020 up to November 2024. (Search URL: <https://scholar.google.com/scholar?cites=487467347958269459&as_sdt=2005&sciodt=0,5&hl=en>.) This search returned 110 articles.

Co-author RC assessed exclusion criteria for these different articles based on the following:

1. Document not in English
2. Document not accessible with our institutional access (Chapman University)
3. Document citing another Park et al. (2020) article (I. S. Park ,  J. U. Kim ,  C. Y. Chan ,  M. Tanaka ,  Y. Tsuchiya ,  H. Nakanotani and  C. Adachi ,  Nat. Commun., 2020,  **11** , 1 —8)
4. Citations were repeats
5. Not a published document (e.g. poster, blog post, patent, etc.)

Five sources were excluded because of item 1. Six sources were excluded because of item 2. Four sources were excluded because of item 3. Ten sources were excluded because of item 4 and three sources were excluded because of item 5.

The citations were then extracted and both RC and LJB labeled each of them according to whether they referred to the behavioral or EEG result. Sources labelled differently were discussed amongst RC and LJB and a final label was determined following this discussion. This happened for 5 sources.

Thirty-one citations were labelled as referring directly to the EEG results (see S1 Table) across twenty-six sources. RC sorted the citations according to the sections or type of source that the citations were in (S1 Table, column 1).
