# Supplementary material for: No evidence for modulation of the readiness potential by respiratory phase during natural breathing
Source: PLoS Biol. 2026 Sep 2;24(9):e3003982. doi: 10.1371/journal.pbio.3003982 (PMC13561358; doi:10.1371/journal.pbio.3003982)
Supplement: Supplementary file 3 [file pbio.3003982.s002.docx]

S1 Table. Table of the 31 citations of the 26 papers referring to Park et al.’s (2020) EEG results up to November 2024.

| **Section / publication type** | **Source** | **Citation** |
| --- | --- | --- |
| **Introduction** | Kluger & Gross 2020 [30] | “Fittingly, a recent study showed that the cortical readiness potential, originating within premotor areas, fluctuates with respiration” |
|  | Nakamura et al. 2022 [32] | “Moreover, Park et al. (2020) found that voluntary motor actions were more frequently initiated during expiration than during inspiration, and cortical readiness potential was coordinated with this behavioral observation.” |
|  | Park et al. 2022 [9] | “More specifically, this study reported that the phase of respiration is coupled with the onset of a voluntary hand movement, and with its neural precursor, the so-called readiness potential (RP), suggesting that the breathing system is involved in a high-level motor cognition.” |
|  | Zhang et al. 2023 [42] | “More interestingly, Park et al (2020) validated that the fluctuations of EEG background activities are not noise, but respiratory signals. For these reasons, our study focuses on the potential of EEG neural signals-based respiratory motion tracking” |
|  | Al et al. 2023 [21] | “How we perceive and engage with the world is influenced by the dynamic relationship between the brain and the rest of the body including respiratory, digestive, and cardiac systems” |
|  | Belli & Fischer 2024 [23] | “For example, the cortical readiness potential varies with respiratory phase, making voluntary actions more frequent during exhalation than inhalation.” |
|  | Engelen et al. 2024 [26] | “The phase of respiration affects […] motor-related neural activity (cortical readiness potential (Park et al., 2020) […]” |
| **Methods** | Park et al. 2022 [9] | “We then tested whether the RP amplitude correlates with the phase of respiration, to replicate a recently reported finding (Park et al., 2020).” |
| **Results** | Park et al. 2022 [9] | “This result shows that the RP amplitude is more negative during the expiration phase compared to the inspiration phase, replicating our previous finding (Park et al., 2020).” |
| **Discussion** | Serré et al. 2021 [38] | “More recently, in a comprehensive study, Park et al. investigated the “readiness potential,” an ERP component consistently found before initiating voluntary actions. Their findings show that voluntary actions frequently occur during exhalation and that the amplitude of readiness potential depends on the respiratory phase, that is, actions are phase coupled to breathing.” |
|  | Kluger & Gross 2021 [13] | “Strong support for this hypothesis comes from a recent study showing that the cortical readiness potential, originating within premotor areas, fluctuates with respiration” |
|  | Pellicano et al. 2021 [33] | “In keeping with this, there is evidence that SMA is involved in respiratory actions” |
|  | Park et al. 2022 [9] | Finally, we observed that the amplitude of the RP was coupled with the phase of respiration (i.e., more negative RP amplitude during the expiration compared to the inspiration period), replicating our recent finding (Park et al., 2020). |
|  | Rahimi et al. 2023 [34] | “To be precise, jogging itself may increase exercise-induced stress insults in a brain with migraine; however, diaphragmatic breathing helps promote stress-response control indices and potentiate cortical readiness before jogging” |
|  | Gavenas et al. 2024 [28] | “One—admittedly speculative—possibility is that these fluctuations are related to fluctuations in arousal, which may explain correlations between the RP and respiration” |
| **Theoretical Paper** | Venerin et al. 2020 [40] | “In several studies, it was demonstrated that activity of neurons as well as activity of co-organized with neurons cells of other tissues (cells of heart, lungs, etc.) changes while passing from one whole-organism result’s achievement to another (see Fig. 1), for example, while switching from approach behaviour to withdrawal behaviour or from cognitive workload to motor workload [86], [87], [88], [89].” |
|  | Vara Sánchez 2020 [39] | “We also know that spontaneous rhythms – but ones which are subject to entrainment – such as breathing, are not only linked to involuntary respiratory-related movements, but also impact voluntary actions by modulating the readiness potential (Park et al. 2020)”  “By taking together Park et al.’s results (2020) suggesting that respiratory-related movements, impact voluntary actions by modulating the readiness potential” |
|  | Safron 2021 [35] | “Further indirect supporting evidence may be found in voluntary actions being more frequently initiated during exhalations, where associated neural dynamics (i.e., readiness potentials) exhibit modulation by respiratory cycles [163].” |
|  | Boyadzhieva & Kayhan 2021 [24] | “Readiness potential amplitudes were also smaller during the expiration phase as compared to inspiration.” |
|  | Waller 2021 [41] | “[…] the RP is modulated by low-level and involuntary respiratory patterns (Park et al. 2020).” |
|  | Criscuolo et al. 2022 [25] | “This idea translates to the brain readiness potential, which displays preferential phase-locking at the end of the inspiration phase, while behavior tends to cluster at the expiration phase [65].” |
|  | Allen et al. 2023 [22] | “Other recent studies report that participants were […] more likely to initiate voluntary actions during inspiration, and that inspiratory phase further enhanced the readiness potential as measured by electroencephalogram (EEG; Park et al., 2020).” |
| **Reviews** | Safron 2021 [35] | “We also begin to see individuals feeling sensations of breath in their bodies (shaded torsos), where exhalations have been associated with action selection and stronger readiness potentials in Libet paradigms (Park et al., 2020)” |
|  | Schurger et al. 2021 [36] | “The breathing cycle, a largely involuntary phenomenon, has been linked to a modulation in the amplitude of the RP [86.]. However, because breathing strongly influences the onset time of spontaneous voluntary movement [86.], it is difficult to determine whether these observations reflect a causal relationship or an incidental one.” |
|  | Jelinčić et al. 2022 [29] | “A different study using EEG reported respiratory phase relating not only to the initiation of voluntary actions, but also to the magnitude of the spontaneous cortical readiness potential arising from premotor areas prior to the action (Park et al., 2020)” |
|  | Dominik et al. 2024 [3] | “The characteristics of the RP are known to be influenced by many factors, such as […] the phase of the respiration cycle (H.-D. Park et al., 2020, Park et al., 2022).” |
| **Theses** | Serré 2022 [37] | “Park and colleagues (2020) showed that readiness potential of voluntary action was coupled to respiratory phases and modulated by the periodical breathing action.”  “[…] the amplitude of readiness potential depends on the respiratory phase […]” |
|  | MacIntyre 2022 [31] | “Recently, Park et al. (2020) demonstrated that participants initiate voluntary actions more frequently during expiration, and that the readiness potential, a neural marker of forthcoming action, is modulated depending on the respiratory phase” |
|  | Gavenas 2023 [27] | “One possibility is that these fluctuations are related to fluctuations in arousal, which may explain correlations between the RP and respiration and pupil dilations before freely timed movement in humans” |
