# Supplementary material for: No evidence for modulation of the readiness potential by respiratory phase during natural breathing
Source: PLoS Biol. 2026 Sep 2;24(9):e3003982. doi: 10.1371/journal.pbio.3003982 (PMC13561358; doi:10.1371/journal.pbio.3003982)
Supplement: Supplementary file 4 [file pbio.3003982.s003.docx]

**S2 Text: Difference in phase of behavioral results**

While we also found a significant coupling of respiratory phase and timing of the button press (permutation test, p = 0.003) in the new data we collected, our distribution of means ranged from the middle of expiration to the middle of inspiration (Fig 1D), while the original means ranged mostly from start to end of expiration (Fig 1A; Fig 1B and 2B in Park et al. 2020). This discrepancy is interesting, and we propose below a few potential explanations for it. We invite future studies to explore the different experimental factors that could influence this coupling and account for this discrepancy.

We would like to point out that while in both datasets the coupling is significant, it is not obvious that there is a large effect size. Assessing the effect size of this coupling is not trivial, and we are not aware of any standard measures for this. However, we propose here a way to quantify how far above chance the strength of the coupling is and show that it is a small but robust effect. Since, at the participant level, this effect is small, we expect that small changes in the experimental factors could account for our dataset differences.

To assess the effect size of the coupling between respiratory phase and the timing of the button press, we used the test statistic from the Hodges-Ajne test for non-uniformity of circular data (also known as the omnibus test) [46]. The test statistic is derived at the participant level and represents the minimum number of events (respiratory phase at time of button press) that can be made to fit in a half circle on the circular scale that ranges from start of expiration to end of inspiration, by rotating a half circle. Essentially this measures how the data is packed into any one half of the circle. It provides an estimate for the uniformity of the data (the smaller the number, the less uniform the data). We took this minimum number, subtracted it from the total number of events and then divided the result by the total number of events to get a proportion of how many events can be on one side of the circle (around 50% here would be expected for perfect uniformity).

Now, because humans spend more time expiring than inspiring [50], it is natural to expect random events to occur more often during expiration. To account for this, at the individual level, we also computed this proportion against the 1000 permutations where the respiratory phase was randomly shifted on each iteration. Note here that while Park et al. (2020) shifted the respiratory signal across the session, we could only do this across concatenated trials for their data due to the data format (the data shared with us was already epoched), however we did this over the entire session for our data. We show that in the original data, participants’ presses are only 3.95% less uniform than for those found in the permutations on average across participants. In our data, this number is even smaller, with the events being only 1.53% less uniform than for permuted data. The distribution of button presses overlaid with the distribution of randomly permuted timepoints (S1 Fig) further exemplifies that the effect is small in Fig 1 or Fig 1B and 2B of the original study because the ratio of expiration to inspiration is skewed to begin with (dark circles on S1 Fig). Note that in our data, the skew is a little earlier during expiration which could indicate a different level of engagement with the task (or potentially, a difference between permuting across the entire session versus across concatenated trials).

Now that we have established that the effect is small, it becomes more plausible that a small change in task demands could cause the difference in the reported findings. There are multiple possible reasons for this. We speculate below on a few possibilities. However, we cannot rule out the possibility that these small differences are due to noise (e.g., sampling differences in the participants) rather than to one or more of the systematic effects that we propose below.

Within Park et al.’s original study, the mean coupling occurred at slightly different phases for participants performing the Kornhuber & Deecke paradigm (with a mean leaning towards the end of expiration, Fig 1B in Park et al. (2020)) versus those performing the Libet task (with a mean more centered near the middle of expiration, Fig 2B in Park et al. (2020)). Based on this slight difference, we speculate that the task or instructions might influence the coupling.

Our task consisted only of Libet-type trials. It is important to note that Park et al’s Libet task only contained W-time trials (75 total per participant) while ours was a mix of W-time and M-time trials (50 each for a total of 100 trials per participant). This difference could be responsible for the different results. Introspective attention is allocated to different processes in M- and W-time trials, as in M-time trials participants are asked to report the timing of their movements while in W-time trials they are asked to report the timing of their intentions. However, there is only a small difference between M- and W-trials coupling in our data (S2A, B Fig).

Another difference of note is that we ran 100 trials per participant while the authors only ran 75. It is possible that the more trials a participant performs the more this coupling shifts. However, the direction of the coupling in our data remains mostly unchanged when looking only at the first 75 trials (S2C Fig).

The inter-trial duration was also very different between our two procedures, with Park et al.’s pre-trial start periods ranging from 4-8 s, while ours was a fixed duration of 1 s. This additional time to relax between trials might have contributed to the difference. A further (minor) difference was the color of the clock’s hand (rotating dot), which was white in our experiment but red in their experiment. Although it is unclear whether it may have also had any effect on the coupling here.

Finally, to speculate a bit, the RP reported is very closely aligned with the timing of the button press in the original data, indicating that their participants were extremely abrupt and spontaneous in their movements (see Fig 3A in Park et al. 2020). On the other hand, the peak of our RP lags a bit behind (Fig 2B), indicating an intertrial variability in the duration it took our participants between initiating the motor response and completing the button press. This is typically remedied by re-aligning to EMG onset (start of motor response), however, in the main manuscript we wanted to keep our analysis as similar to Park et al’s (2020) as possible, therefore we did not perform such realignment in the main figure, although we show (S2D Fig) that even when such realignment is performed, the coupling remains at the same phase of breathing. This delay in the RP peak could be caused by differing instructions (although note that we asked our participants to press abruptly and spontaneously and they had their fingers resting on the button, ready to press at all times) or by the sensitivity of the button (if the button is hard to press it may take longer for the action to be registered compared to a very sensitive button). We do not expect this difference (of ~200 ms in the peak of the RP) to be the cause for such a difference in phase (our average respiratory rate was 0.31 Hz, therefore 200 ms only covers ~2/5^th^ of a respiratory bin). Nevertheless, to be sure that it had no effect in our data, the activity from the EMG was band-pass filtered between 20 and 200 Hz, and then we low-pass filtered the absolute value of the resulting signal at 10 Hz. Within the last 0.5 s of each trial, when the 97.5^th^ percentile of the rectified EMG signal from the 4 s preceding the button press was crossed for the first time, the event was realigned to this threshold crossing (S2D Fig).

While we are not certain of the cause of the discrepancy between respiratory coupling phase in the original results and in our data, we did find a significant coupling of breathing with behavior – replicating Park et al.’s behavioral result. We invite future studies to explore the different factors that drive and modulate this coupling of respiration and movement initiation.
