# Supplementary material for: No evidence for modulation of the readiness potential by respiratory phase during natural breathing
Source: PLoS Biol. 2026 Sep 2;24(9):e3003982. doi: 10.1371/journal.pbio.3003982 (PMC13561358; doi:10.1371/journal.pbio.3003982)
Supplement: Supplementary file 5 [file pbio.3003982.s006.docx]

**S3 Text: Outliers and normalization**

Besides correcting the binning approach, we identified other methodological issues that we address here. These have led to our use of no normalization in our corrected binning method (Fig 1C, F, I). We describe here how these different methods affect the data. We note that fixing the binning method is enough to render the EEG results (respiration-RP coupling) no longer significant, regardless of the parameters selected for the steps described below.

Firstly, the confounder of the behavioral result, when unaccounted for, should bias the coupling analysis’ mode to align with the preferred respiratory phase for movement initiation. Indeed, if the RP is most negative right before movement onset, and movement onset occurs mostly during the end of expiration, then the EEG signal at the end of expiration should be most negative. However, this is not the case: the mode occurs during the start of inspiration (Fig 1B).

We argue this is both due to outliers and due to the choice of normalization. We identified large outliers in the data that alter the results in significant ways. In the original data, removing the single most extreme trial shifts the mode of the distribution by one full bin (S3B Fig). Removing the single most extreme participant also shifts the mode of the distribution by one bin but also flattens it (S3C Fig). Another important note is that when applying outlier rejection to our corrected binning method, the trending significance is abolished for the original data (Fig 1C) with a p-value of 0.237 with 2 outliers detected using the 3-standard deviation method used in Park et al. (2020) to perform outlier rejection during preprocessing of EEG. Using the interquartile range method consisting of removing participants with an MI larger than the third quartile plus 1.5 times the interquartile range, we obtain p = 0.591, with 5 outliers detected.

**S3 Fig.** Impact of outliers on the amplitude of the RP as a function of six equally sized bins of the respiratory phase using the original binning method for n=52 participants. Error bars represent the SEM. A no outliers rejected. B one single most extreme outlier trial rejected. C one single most outlier participant rejected. The data and code used to reproduce this figure are available via OSF and GitHub; some of the original data are available only upon request from the authors of Park et al. (2020). See the Data Availability Statement for details.

One of the reasons outlier rejection impacts the EEG results using the original binning so much (S3 Fig) is because the normalization step was not appropriate for the given data. The data were normalized within each trial by dividing all six bins’ values by the sum of the bins (henceforth “sum division”). This normalization is a requirement for the modulation index employed here [44], but unfortunately it only works for values that are positive. For instance, should the 6 bins comprise the following positive and negative values: 2.1, -1.5, 3.2, -2.8, -0.7 and -0.4; then clearly the fourth item, -2.8, is the smallest value. However, sum division would yield the following normalized values: -21, 15, -32, 28, 7 and 4. Now, the third item, -32 (normalized version of 3.2) is now the smallest. Note that a small change in this example, changing the first item, 2.1, into 2.7, would now yield the following series: 1.35, -0.75, 1.6, -1.4, -0.35 and -0.2. This small change thus had dramatic consequences on both the sign and magnitude of each value. This issue is typically not a problem when using the envelope of a signal which the MI was designed for [44], however due to the nature of the RP (it is a very slow signal with a low signal-to-noise ratio and arguably no cyclicity) it is not standard to extract the envelope of the RP. Instead, the authors used the non-enveloped EEG signal and added an arbitrary positive value (500) to each bin prior to normalizing when computing their modulation index but not when plotting the data (Fig. 3b in Park et al.). Adding this 500 ensured that none of the signal was ever negative such that all values were positive prior to normalizing. In our example above, adding 5 to all values bypasses the above-mentioned issues as the two different series would become 0.237, 0.117, 0.274, 0.074, 0.145 and 0.154 for the first one and 0.252, 0.115, 0.269, 0.072, 0.141 and 0.151 for the second one. Both would preserve the order of smallest to largest values and a comparable magnitude. However, they only added the 500 when computing the test statistic. So, while this does not invalidate the significance of the coupling, it does invalidate any qualitative conclusions that could be drawn visually from the graph (i.e. the peak of the coupling is not really at the start of inspiration as the plot suggests).

Using this normalization technique on vectors that contain both positive and negative values might flip the sign of the data if the data were negative, or even worse, it might create arbitrarily large values if the sum were close to zero, as shown in the example above. The creation of arbitrarily large values here is what explains the consequential role of single outliers when using this normalization technique.

In our approach we did not perform any normalization (Fig 1C, F and I) because our approach did not require any. The RP does not require normalization to be compared across participants. The normalization was a required step for computing the MI, however, in order to compute a pseudo-probability density function [44]. Note that, while for display purposes we did not perform normalization, for computing the MI we did (using the same +500 trick as the authors).

We show here how the choice of normalization (sum normalization as described above or no normalization) affects the data. Using the original binning, the mode of the coupling lands at the start of inspiration for sum-normalization (S4A Fig) while without normalizing, the mode (most negative RP) is at the middle of expiration, where it is expected to be given the behavioral confounder (S4B Fig). Using the corrected binning, the sum-normalization yields arbitrary positive and negative values for each bin (S4C Fig), while no normalization results in a more or less uniform distribution across peaks (negative because the RP is negative; S4D Fig). The RP amplitude for each participant at each trial is measured on the same scale (in µV) and therefore does not necessitate the use of normalization.

Both the outliers and normalization steps explain why the peak of the coupling did not align with the phase of the behavioral confounder.
