# Supplementary material for: No evidence for modulation of the readiness potential by respiratory phase during natural breathing
Source: PLoS Biol. 2026 Sep 2;24(9):e3003982. doi: 10.1371/journal.pbio.3003982 (PMC13561358; doi:10.1371/journal.pbio.3003982)
Supplement: Peer review history

# Editor report: Decision Letter 0

**DOI:** 10.1371/journal.pbio.3003982.r001

**Authors:** Lucas Smith

3 Jun 2026

Dear Dr Jeay-Bizot,

Thank you for submitting your manuscript entitled "No evidence for modulation of the readiness potential by respiratory phase" for consideration as a Short Report by PLOS Biology. Your manuscript has been assessed as a revision, in response to the previous rounds of review at a different journal, under our portable peer review policy. We have discussed your manuscript and the previous rounds of review with an Academic Editor with relevant expertise, and I am pleased to let you know that we think that your paper would be suitable for publication at PLOS Biology after relatively minor revisions, and without the need for additional rounds of peer review.

While we think that your revision has satisfactorily addressed the previous reviewer comments, we do have a few editorial suggestions that we would like you to address in a last, short revision before we are able to formally accept yours study. However, before we can invite the next round of revision we need you to complete your submission by providing the metadata that is required for full assessment. To this end, please login to Editorial Manager where you will find the paper in the 'Submissions Needing Revisions' folder on your homepage. Please click 'Revise Submission' from the Action Links and complete all additional questions in the submission questionnaire.

Once your full submission is complete, your paper will undergo a series of checks. After your manuscript has passed the checks it will be passed back to me, and I will then invite you to revise your study and will share our editorial requests.

To provide the metadata for your submission, please Login to Editorial Manager (https://www.editorialmanager.com/pbiology) within two working days, i.e. by Jun 05 2026 11:59PM.

Feel free to email us at plosbiology@plos.org if you have any queries relating to your submission.

Kind regards,

Luke

Lucas Smith, Ph.D.

Senior Editor

PLOS Biology

lsmith@plos.org

# Editor report: Decision Letter 1

**DOI:** 10.1371/journal.pbio.3003982.r002

**Authors:** Lucas Smith

5 Jun 2026

Dear Dr Jeay-Bizot,

Thank you again for submitting your manuscript "No evidence for modulation of the readiness potential by respiratory phase" for consideration as a Short Report at PLOS Biology, under our portable peer review policy. As mentioned in my previous email, your study has been assessed by the PLOS Biology editorial staff and an Academic Editor with relevant expertise, as a revision in response to reviews from a different journal. Overall, we think your study has addressed the previous reviewer comments and we are likely to accept your study.

However, before we can editorially accept your manuscript, we do have a few editorial comments, and data/policy requests, which I detail below my signature. We would like to invite you to address these points in a revision that we anticipate should not take you very long. We will then assess your revised manuscript and your response to the reviewers' comments with our Academic Editor aiming to avoid further rounds of peer-review, although there is a small chance that we might need to consult with the reviewers, depending on the nature of the revisions.

In addition to these revisions, you may need to complete some formatting changes, which you will receive in a follow up email. A member of our team will be in touch with a set of requests shortly. If you do not receive a separate email within a few days, please assume that checks have been completed, and no additional changes are required.

We expect to receive your revised manuscript within 1 month. Please email us (plosbiology@plos.org) if you have any questions or concerns, or would like to request an extension.

At this stage, your manuscript remains formally under active consideration at our journal; please notify us by email if you do not intend to submit a revision so that we withdraw the manuscript.

**IMPORTANT - SUBMITTING YOUR REVISION**

Your revisions should address the specific points made by each reviewer. Please submit the following files along with your revised manuscript:

1. A 'Response to Reviewers' file - this should detail your responses to the editorial requests, present a point-by-point response to all of the reviewers' comments, and indicate the changes made to the manuscript.

*NOTE: In your point-by-point response to the reviewers, please provide the full context of each review. Do not selectively quote paragraphs or sentences to reply to. The entire set of reviewer comments should be present in full and each specific point should be responded to individually.

You should also cite any additional relevant literature that has been published since the original submission and mention any additional citations in your response.

2. In addition to a clean copy of the manuscript, please also upload a 'track-changes' version of your manuscript that specifies the edits made. This should be uploaded as a "Revised Article with Changes Highlighted " file type.

*Resubmission Checklist*

When you are ready to resubmit your revised manuscript, please refer to this resubmission checklist: https://plos.io/Biology_Checklist

To submit a revised version of your manuscript, please go to https://www.editorialmanager.com/pbiology/ and log in as an Author. Click the link labelled 'Submissions Needing Revision' where you will find your submission record.

Please make sure to read the following important policies and guidelines while preparing your revision:

*Published Peer Review*

Please note while forming your response, if your article is accepted, you may have the opportunity to make the peer review history publicly available. The record will include editor decision letters (with reviews) and your responses to reviewer comments. If eligible, we will contact you to opt in or out. Please see here for more details:

https://blogs.plos.org/plos/2019/05/plos-journals-now-open-for-published-peer-review/

*PLOS Data Policy*

Please note that as a condition of publication PLOS' data policy (http://journals.plos.org/plosbiology/s/data-availability) requires that you make available all data used to draw the conclusions arrived at in your manuscript. If you have not already done so, you must include any data used in your manuscript either in appropriate repositories, within the body of the manuscript, or as supporting information (N.B. this includes any numerical values that were used to generate graphs, histograms etc.). For an example see here: http://www.plosbiology.org/article/info%3Adoi%2F10.1371%2Fjournal.pbio.1001908#s5

*Blot and Gel Data Policy*

We require the original, uncropped and minimally adjusted images supporting all blot and gel results reported in an article's figures or Supporting Information files. We will require these files before a manuscript can be accepted so please prepare them now, if you have not already uploaded them. Please carefully read our guidelines for how to prepare and upload this data: https://journals.plos.org/plosbiology/s/figures#loc-blot-and-gel-reporting-requirements

*Protocols deposition*

To enhance the reproducibility of your results, we recommend that if applicable you deposit your laboratory protocols in protocols.io, where a protocol can be assigned its own identifier (DOI) such that it can be cited independently in the future. Additionally, PLOS ONE offers an option for publishing peer-reviewed Lab Protocol articles, which describe protocols hosted on protocols.io. Read more information on sharing protocols at https://plos.org/protocols?utm_medium=editorial-email&utm_source=authorletters&utm_campaign=protocols

Thank you again for your submission to our journal. We hope that our editorial process has been constructive thus far, and we welcome your feedback at any time. Please don't hesitate to contact us if you have any questions or comments.

Sincerely,

Luke

Lucas Smith, Ph.D.

Senior Editor

PLOS Biology

lsmith@plos.org

----------------------------------------------------------------

EDITORIAL REQUESTS:

1) FORMAT: We understand that your original submission needed to be very concise, and that you have kept it that way while submitting to PLOS Biology, given that the previous reviewers were largely supportive of the previous version. While we appreciate your instinct, we do think that the paper should be fleshed out, as this will help the non-familiar reader and will broaden the appeal of your study. Below are a few specific suggestions:

a. Currently the framing of the study and findings are quite deeply embedded in the context of the two papers which your results challenge. However, I think it would be helpful to adjust the introduction and discussion a bit, to provide more of the broader context and background details (for example, what is readiness potential? why is it important? in what other contexts has breathing been linked to neuronal activity, behavior etc? do your findings prompt a re-evaluation of that work too? etc).

b. Along these lines, I also found your results and methods sections to be a bit thin on details. As just one example, your results start discussing how 'we reproduced...' - without describing what analysis you did, or in what dataset. As an example from the methods, you discuss how you 'analyzed three datasets' - what types of datasets were these? what type of data? etc... In general, I think both of these sections could be fleshed out to help bring the reader along, and to ensure that people understand what was done and why.

c. I did notice that your SI includes a lot of additional details and discussion, and I wonder if one approach could be to integrate many of those details into the main paper. I think many of the details that you include in the SI would be reasonable to put in the methods section or in the results and discussion. Please also note that any citations that are included in the SI must also be included in the main manuscript (and reference list), as otherwise these will not be indexed.

d. As a last point about fleshing the study out - we understand that since your paper was under consideration at a different journal that several relevant papers have come out on this topic. While we understand that your manuscript already mentions these, we think the discussion of those papers could be expanded a bit, to make sure your findings are presented in the most relevant, and up-to date context.

2) TITLE: After discussing your paper with my colleagues, we did wonder if it would be a good idea to tweak the title slightly, in light of recent literature. If you agree, we would suggest you update the title to:

'No evidence for modulation of the readiness potential by respiratory phase during natural breathing'

^we are happy to discuss further.

3) ABSTRACT: Please note that it is PLOS Biology policy to clearly state in the abstract what model organisms was examined in the study. Please update the abstract accordingly.

4) ETHICS STATEMENT: Please update your ethics statement to include the approval number for your protocol approved by Chapman University's IRB. Please also indicate whether your study was conducted in accordance with the principles laid out in the Declaration of Helsinki.

5) DATA and CODE: Thank you for providing the raw data on OSF, and the process and simulated data on GitHub (including with a zenodo DOI). These largely meet our requirements, but:

a. please add a sentence to every relevant figure legend, pointing readers to the underlying data.

b. please update your data availability statement in our editorial manager system, to indicate that the code can also be found on your GitHub deposition.

# Author comment: Author response to Decision Letter 2

**DOI:** 10.1371/journal.pbio.3003982.r003

20 Jul 2026

Attachment Submitted filename: Response_to_Reviewers.pdf

# Editor report: Decision Letter 2

**DOI:** 10.1371/journal.pbio.3003982.r004

**Authors:** Lucas Smith

30 Jul 2026

Dear Dr Jeay-Bizot,

Thank you for your patience while we considered your revised manuscript "No evidence for modulation of the readiness potential by respiratory phase during natural breathing" for publication as a Short Report at PLOS Biology. This revised version of your manuscript has been evaluated by the PLOS Biology editors and the Academic Editor.

We are largely satisfied by the revision and think the presentation of the study is now improved. However we did spot a few minor issues and we think these should be addressed in a last, short revision. I have detailed these points below my signature.

As you address these items, please take this last chance to review your reference list to ensure that it is complete and correct. If you have cited papers that have been retracted, please include the rationale for doing so in the manuscript text, or remove these references and replace them with relevant current references. Any changes to the reference list should be mentioned in the cover letter that accompanies your revised manuscript.

In addition to these revisions, you may need to complete some formatting changes, which you will receive in a follow up email. A member of our team will be in touch with a set of requests shortly. If you do not receive a separate email within a few days, please assume that checks have been completed, and no additional changes are required.

We expect to receive your revised manuscript within two weeks.

To submit your revision, please go to https://www.editorialmanager.com/pbiology/ and log in as an Author. Click the link labelled 'Submissions Needing Revision' to find your submission record. Your revised submission must include the following:

- a cover letter that should detail your responses to any editorial requests, if applicable, and whether changes have been made to the reference list

- a Response to Reviewers file that provides a detailed response to the reviewers' comments (if applicable, if not applicable please do not delete your existing 'Response to Reviewers' file.)

- a track-changes file indicating any changes that you have made to the manuscript.

NOTE: If Supporting Information files are included with your article, note that these are not copyedited and will be published as they are submitted. Please ensure that these files are legible and of high quality (at least 300 dpi) in an easily accessible file format. For this reason, please be aware that any references listed in an SI file will not be indexed. For more information, see our Supporting Information guidelines:

https://journals.plos.org/plosbiology/s/supporting-information

*Published Peer Review History*

Please note that you may have the opportunity to make the peer review history publicly available. The record will include editor decision letters (with reviews) and your responses to reviewer comments. If eligible, we will contact you to opt in or out. Please see here for more details:

https://plos.org/published-peer-review-history/

*Press*

Should you, your institution's press office or the journal office choose to press release your paper, please ensure you have opted out of Early Article Posting on the submission form. We ask that you notify us as soon as possible if you or your institution is planning to press release the article.

*Protocols deposition*

To enhance the reproducibility of your results, we recommend that if applicable you deposit your laboratory protocols in protocols.io, where a protocol can be assigned its own identifier (DOI) such that it can be cited independently in the future. Additionally, PLOS ONE offers an option for publishing peer-reviewed Lab Protocol articles, which describe protocols hosted on protocols.io. Read more information on sharing protocols at https://plos.org/protocols?utm_medium=editorial-email&utm_source=authorletters&utm_campaign=protocols

Please do not hesitate to contact me should you have any questions.

Sincerely,

Luke

Lucas Smith, Ph.D.

Senior Editor

lsmith@plos.org

PLOS Biology

------------------------------------------------------------------------

COMMENTS FROM THE ACADEMIC EDITOR

I have reviewed the revised manuscript and am happy with the authors’ work. They have addressed the requested revisions well, and the paper is now much clearer and better contextualised. I do not have any remaining scientific concerns and support moving towards acceptance without further peer review.

I noticed a few minor technical and presentational issues, particularly some inconsistencies between the revised manuscript and Supporting Information, as well as an apparent transcription error in one of the reported credible intervals. These should be straightforward to resolve editorially and do not affect my overall assessment.

* The Supporting Information does not seem to have been fully updated alongside the manuscript. It still has the old title and uses “Supplementary Notes 1–4,” whereas the manuscript lists “S1 Text–S3 Text.” Supplementary Note 2 also remains in the SI even though the response says that material was moved into the main Discussion, and there is a visible “Formatted: Highlight” marker. Supplementary Note 3 reports the behavioral result as p < 0.001 using an MI permutation test, while the main text reports p = 0.003 from the Hodges–Ajne analysis.

* There is an apparent transcription error in the Bayesian results. For the inspiration-versus-expiration comparison in the original dataset, the posterior median is reported as −0.136, but the 95% credible interval is given as [−0.128, 0.402], which does not contain the median. The corresponding interval for the new dataset is also missing a comma.

* A few smaller points: the ethics statement is not placed under the requested subheading at the beginning of the Methods; the normalized values in Fig 1B, E and H are labelled in µV despite being dimensionless; and there is a typo in the Methods, “asked to repor where.”

OTHER EDITORIAL REQUESTS (from me):

1 - Please update the supplemental figure legends to include a reference to where the underlying data can be found.

2 - I noticed that you still have references in the supplemental materials that are not included in the main manuscript (in the 'impact on the field' section). As noted, we do not allow this, as references that are only presented in the SI will not be indexed.

I think one possible solution would be to just include those references at the same time that you refer readers to the SI text, where they appear. Ex I think they could be added naturally to the sentence 'The notion of a causal relation between respiration and the RP has been extensively repeated in the literature (see S1 Text --references ____)'

3 - Given the issues spotted by the Academic Editor and the substantial re-organization of your paper that occurred in the revision, we encourage you to given your manuscript a thorough once-over to ensure there no other inconsistencies or errors were introduced during the revision. If you spot any other areas that require substantial changes, please be sure to describe these in the cover letter during your next resubmission.

# Editor report: Decision Letter 3

**DOI:** 10.1371/journal.pbio.3003982.r005

**Authors:** Lucas Smith

18 Aug 2026

Dear Dr Jeay-Bizot,

Thank you for the submission of your revised Short Reports "No evidence for modulation of the readiness potential by respiratory phase during natural breathing" for publication in PLOS Biology and thank you for addressing our last editorial requests in this revision. On behalf of my colleagues and the Academic Editor, Micah Allen, I am pleased to say that we can in principle accept your manuscript for publication, provided you address any remaining formatting and reporting issues. These will be detailed in an email you should receive within 2-3 business days from our colleagues in the journal operations team; no action is required from you until then. Please note that we will not be able to formally accept your manuscript and schedule it for publication until you have completed any requested changes.

Please take a minute to log into Editorial Manager at http://www.editorialmanager.com/pbiology/, click the "Update My Information" link at the top of the page, and update your user information to ensure an efficient production process.

PRESS

We frequently collaborate with press offices. If your institution or institutions have a press office, please notify them about your upcoming paper at this point, to enable them to help maximise its impact. If the press office is planning to promote your findings, we would be grateful if they could coordinate with biologypress@plos.org. If you have previously opted in to the early version process, we ask that you notify us immediately of any press plans so that we may opt out on your behalf.

We also ask that you take this opportunity to read our Embargo Policy regarding the discussion, promotion and media coverage of work that is yet to be published by PLOS. As your manuscript is not yet published, it is bound by the conditions of our Embargo Policy. Please be aware that this policy is in place both to ensure that any press coverage of your article is fully substantiated and to provide a direct link between such coverage and the published work. For full details of our Embargo Policy, please visit http://www.plos.org/about/media-inquiries/embargo-policy/.

Thank you again for choosing PLOS Biology for publication and supporting Open Access publishing. We look forward to publishing your study.

Sincerely,

Lucas Smith, Ph.D.

Senior Editor

PLOS Biology

lsmith@plos.org
